# The relationship between executive functioning and addictive behavior: new insights from a longitudinal community study

**DOI:** 10.1007/s00213-022-06224-3

**Published:** 2022-10-03

**Authors:** Anja Kräplin, Mohsen Joshanloo, Max Wolff, Klaus-Martin Krönke, Thomas Goschke, Gerhard Bühringer, Michael N. Smolka

**Affiliations:** 1 Work Group Addictive Behaviors, Risk Analysis and Risk Management, Faculty of Psychology, Technische Universität Dresden, Chemnitzer Straße 46, D-01187 Dresden, Germany; 2 Department of Psychology, Keimyung University, Daegu, South Korea; 3 Department of Psychiatry, Technische Universität Dresden, Dresden, Germany; 4 MIND Foundation, Berlin, Germany; 5 Charité – Universitätsmedizin Berlin, corporate member of Freie Universität Berlin and Humboldt-Universität zu Berlin, Department of Psychiatry and Psychotherapy, Campus Charité Mitte, Berlin, Germany; 6 IFT Institut für Therapieforschung, Munich, Germany; 7 Department of Clinical Research, Faculty of Health, University of Southern Denmark, Odense, Denmark

**Keywords:** Substance use disorders, Behavioral addictions, Executive functions, Cognitive control, Self-control

## Abstract

**Rationale:**

Although there is evidence that impaired executive functioning plays a role in addictive behavior, the longitudinal relationship between the two remains relatively unknown.

**Objectives:**

In a prospective-longitudinal community study, we tested the hypothesis that lower executive functioning is associated with more addictive behavior at one point in time and over time.

**Methods:**

Three hundred and thirty-eight individuals (19–27 years, 59% female) from a random community sample were recruited into three groups: addictive disorders related to substances (*n* = 100) or to behaviors (*n* = 118), or healthy controls (*n* = 120). At baseline, participants completed nine executive function tasks from which a latent variable of general executive functioning (GEF) was derived. Addictive behavior (i.e., quantity and frequency of use, and number of DSM-5 criteria met) were assessed using standardized clinical interviews at baseline and three annual follow-ups. The trajectories of addictive behaviors were examined using latent growth curve modeling.

**Results:**

At baseline, we found weak to no evidence of an associations between GEF and addictive behavior. We found evidence for an association between a lower GEF at baseline and a higher increase in the quantity of use and a smaller decrease in frequency of use over time, but no evidence for an association with an increase in the number of DSM-5 criteria met.

**Conclusions:**

Lower EFs appear to lead to a continuing loss of control over use, whereas addictive disorders may develop secondarily after a long period of risky use. Previous etiological models assuming lower EF as a direct vulnerability factor for addictive disorders need to be refined.

**Supplementary Information:**

The online version contains supplementary material available at 10.1007/s00213-022-06224-3.

## Introduction

Executive functions (EFs) are higher level cognitive processes that control and regulate goal-directed behavior (Friedman and Miyake 2017). Inter-individual variability in EFs is generally assumed to explain the onset and course of addictive behavior (Brand et al. 2019; Stephan et al. 2017). Within the scope of this paper, addictive behavior is broadly defined, including quantity and frequency of use (Rehm et al. 2013) and the level of addictive disorder severity (American Psychiatric Association (APA) 2013). We tested the hypothesis of whether relatively low EF abilities are associated with a higher level of addictive behavior both cross-sectionally and longitudinally.

Over the last three decades, several literature reviews and corresponding theoretical models have assumed that impaired EFs are important key factors in the development of addictive behavior, as it leads to an insufficient top-down regulation of behavior concerning long-term goals (e.g., Brand et al. 2019; Finn 2002; Goldstein and Volkow 2011; Goschke 2014; Nigg et al. 2004; Pihl et al. 1990; Tang et al. 2015). Consistent with terminology proposed 20 years ago by Metcalfe and Mischel (1999), but still relevant as discussed in a recent literature review by Friedman and Robbins (2022), the focus of this paper is on “cold” EF tasks such as the Stroop task or the n-back task and not on “hot” tasks such as delay discounting tasks.

Supporting the assumptions from the theoretical models presented above, individuals with substance-related or behavioral addictions demonstrate impaired performance and aberrant brain activity compared to healthy controls in “cold” EF tasks in cross-sectional studies (for meta-analyses, see Chowdhury et al. 2017; Smith et al. 2014; Stephan et al. 2017). While overall group differences are evident, the size of group differences varies widely. The heterogeneity of the results may be due to different sample characteristics such as age, clinical status, comorbidity, gender distribution, or the type of addictive behavior (Liu et al. 2019; Smith et al. 2014).

The heterogeneity of results may also be due to methodological aspects concerning the tasks used to assess EFs. Recent research has indicated that EF tasks may not be adequate to capture individual differences because the within-person effects mask between-person variance (Rouder et al. 2019; Rouder and Haaf 2019). Moreover, the often complex behavioral tasks require several processes and are generally associated with low reliability (Miyake et al. 2000). Latent variable modeling of EF tasks alleviates task-impurity and reliability problems by extracting latent factors reflecting what is common among the EF tasks (Miyake et al. 2000, p.54) and by taking measurement errors into account (Goschke 2014; Gustavson et al. 2017). According to these models, inter-individual variability in different EFs depends to a considerable extent on a common factor referred to as general executive functioning (GEF; Friedman and Miyake 2017; Wolff et al. 2020). In a recent analysis within our project, we also found evidence for such a common GEF factor (Wolff et al. 2020).

In this paper, we aimed at testing cross-sectional and longitudinal relationships between the common GEF factor and the trajectories of addictive behaviors. We assume that lower GEF is related to higher impulsivity and externalizing behavior, and may thus increase use and the addictive disorder severity over time (Gustavson et al. 2017; Young et al. 2009).To get closer to causal conclusions, longitudinal data are most needed (De Wit 2009; Verdejo-Garcia et al. 2008). It is important that these data should come from community samples, as clinical samples have very specific characteristics (e.g., lower executive function due to neurological damage; Naim-Feil et al. 2013) that would limit the generalizability of results. To date, several high-quality longitudinal studies with mostly community-based samples tested the hypothesis that lower EFs predict the development of addictive behavior (Fernie et al. 2013; Gustavson et al. 2017; Khurana et al. 2013; Nigg et al. 2006; Peeters et al. 2015; Tapert et al. 2002; Wilens et al. 2011; Wong et al. 2006). These studies differed in several aspects, of which we would like to highlight three. First, these studies differed in terms of the age studied, ranging from predicting substance use in adolescence with childhood EFs (Nigg et al. 2006; Wong et al. 2006) to predicting substance use in young adults with EFs from adolescence (Khurana et al. 2013; Tapert et al. 2002). We addressed the research question of whether lower EFs also predict the course of addictive behavior in a young non-clinical adult sample from the community.

Second, previous longitudinal studies also differed in the operationalization of EFs, ranging from self-reports of behavioral control (Wong et al. 2006) to one EF task (Khurana et al. 2013; Nigg et al. 2006; Tapert et al. 2002) to a battery of different EF tasks (Fernie et al. 2013; Peeters et al. 2015; Wilens et al. 2011). Our aim was to apply a latent variable approach to model GEF and predict the development addictive behavior, which has only been done in a single study to date (Gustavson et al. 2017).

Third, previous longitudinal studies differed in their outcomes, ranging from using various combinations of addiction outcomes at one or two specific time points (Fernie et al. 2013; Gustavson et al. 2017; Nigg et al. 2006; Peeters et al. 2015; Tapert et al. 2002; Verdejo-Garcia et al. 2008; Wilens et al. 2011) to trajectories of one outcome such as alcohol use over time (Khurana et al. 2013; Wong et al. 2006). We aimed to examine the individual trajectories of various addictive behaviors to better understand the dynamic relationship between EFs and addictive behavior over time.

Regarding addictive behavior, we had no clear hypotheses that EFs may be differently related to substance- and non-substance-related outcomes. Following various models of addictive behavior such as the Model of the Addiction Syndrome (Shaffer et al. 2004), the Component Model of Addiction (Griffiths 2005), or the Model of the Common Liability to Addiction (Vanyukov et al. 2012), we assumed that many commonalities exist between the various manifestations of addictive behavior (e.g., loss of control over time and amount) and that these commonalities reflect a shared etiology. Therefore, we tested our hypotheses across various forms of addictive behavior. To still contribute to the debate about whether all addictive disorders are expressions of a common clinical syndrome (Petry et al. 2014; Shaffer et al. 2004), we exploratory compared substance- and non-substance-related addictive behavior. We also aimed to clarify the role of EFs separately for (non-pathological and pathological) use and addictive disorders. This aim builds on previous findings from a meta-analysis and from our project that support the need to distinguish between consumption and addictive disorder severity when analyzing cross-sectional and longitudinal relationships between “hot” cognitive control and addictive behavior (Amlung et al. 2017; Kräplin et al. 2020). In the present work, we aimed to investigate whether these differences in the predictive value also apply to “cold” cognitive control.

Despite heterogeneity, all studies consistently reported evidence for the hypothesis that lower EF predicts substance use and substance-related problems, but not addictive disorders. Moreover, these studies showed evidence of an association with EFs for various addictive behaviors (and not with a particular behavior, such as alcohol consumption). We aimed to test this hypothesis beyond previous studies by operationalizing EFs as a latent variable from multiple tasks and predicting separate trajectories for (substance-related and non-substance-related) use and symptoms of addictive disorder in young adults. Specifically, we hypothesized that lower GEF characterizes individuals with substance-related and behavioral addictions compared to healthy controls (Hypothesis 1) and that lower GEF would be associated with more substance-related and non-substance-related addictive behavior cross-sectionally (Hypothesis 2) and longitudinally (Hypothesis 3), i.e., higher quantity and frequency of use and an increased number of diagnostic criteria met.

## Methods

### Design and procedure

Data were collected as part of the prospective-longitudinal community study “Volitional dysfunction in self-control failures and addictive behaviors” within the Collaborative Research Centre SFB 940 “Volition and Cognitive Control” at Technische Universität Dresden, Germany (study protocol at ClinicalTrial.gov NCT04498988 and on the OSF at https://osf.io/yu5rm/). The procedure at baseline consisted of (first) a clinical assessment in an interview room, (second) ecological momentary assessment (EMA) of self-control failures in daily life, (third) an experimental task battery in a laboratory (assessing EF abilities and decision-making), and (fourth) functional magnetic resonance imaging (fMRI). Three annual clinical follow-ups were scheduled according to the date of the last of the four baseline sessions, i.e., the fMRI session. This was appropriate for all participants with the exception of six participants, for whom the fMRI session took place after the first baseline clinical assessment with a delay of more than 1 year. For these six participants, we re-allocated the outcome values to later follow-up waves according to the baseline clinical assessment instead of the baseline fMRI session (e.g., data from a 1-year follow-up that took place 1 year after the participant’s fMRI session, but 2 years after the participant’s baseline assessment, was re-allocated to the 2-year follow-up). As described below, smaller individual variations in annual follow-up times were considered in the analyses. Measures from the annual clinical assessments and the baseline EF tasks are described in detail below. Previous publications from this study focused on the prediction of use and addictive disorder severity by impulsive decision making (Kräplin et al. 2020) and the relation between self-control failures with the fMRI and EMA data (Krönke et al. 2021; Krönke et al. 2018; Krönke et al. 2020a; Krönke et al. 2020b; Wolff et al. 2020, 2016).

### Recruitment and participants

Between 2013 and 2016, random samples of 18,000 inhabitants aged between 19 and 27 were taken from the registration office files of Dresden, Germany, and invited by post to participate in the study. A community sample was chosen because we were interested in the generalizability of our results to the population, which consists mainly of non-clinical cases. The age range was chosen to allow for large changes in addictive behavior outcomes (Wagner and Anthony 2002; Wittchen et al. 2008a) while minimizing the influence of neurodevelopmental processes (Casey and Jones 2010). Of all invited inhabitants, 1856 (10.3%) responded to our invitation letter. Respondents were more likely to be younger and female compared to non-respondents (Table S1 in the online supplemental materials). At baseline, we wanted to achieve a high internal validity of our cross-sectional comparison of latent EFs between pure substance-related (i.e., with no lifetime behavioral addiction) and pure behavioral addictions (i.e., with no lifetime substance-related addiction). Therefore, the included participants had to meet the criteria for one of the following three groups:

1. Legal substance use disorder (SUD) group: in the past 12 months, participants met two or more criteria for an alcohol and/or tobacco use disorder according to the fifth edition of the Diagnostic and Statistical Manual of Mental Disorders (DSM-5; APA 2013), but had no lifetime behavioral addiction (i.e., less than two symptoms of one behavioral addiction).
2. Behavioral addiction (BA) group: in the past 12 months, participants met two or more criteria for a DSM-5 gambling or addictive disorders related to internet use, gaming, or shopping assessed with adapted criteria according to the DSM-5 SUD criteria, but had no lifetime SUD (i.e., less than two symptoms of one SUD).
3. Control group: participants had no lifetime BA or SUD diagnoses.

**Fig. 1:**
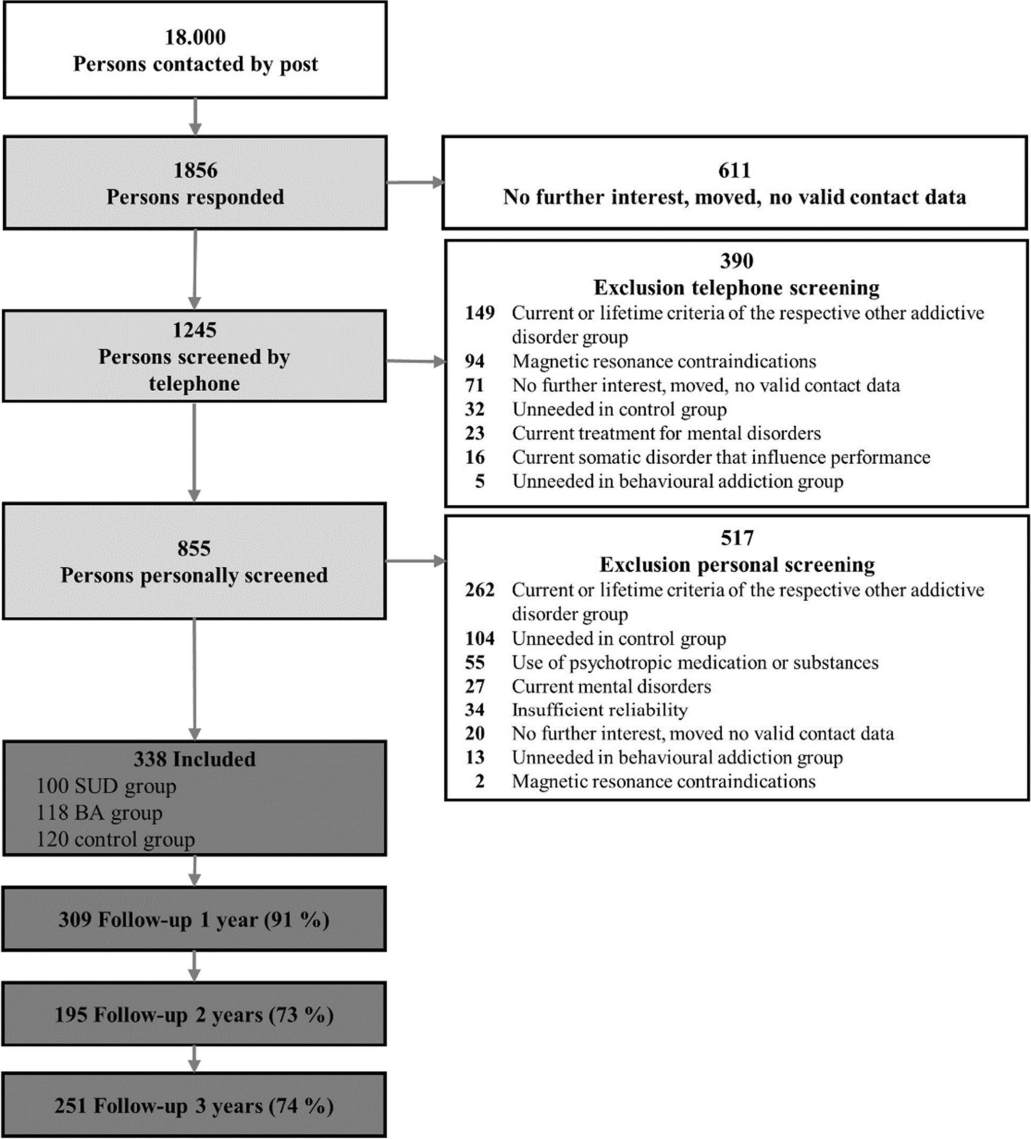
Participant flowchart with numbers and reasons for inclusion and exclusion

Only a subset of addictive behaviors was included to achieve a relatively homogenous sample. To achieve a homogenous group definition, we also defined BA as meeting 2 or more of the 11 criteria adapted from SUD. Exclusion criteria for all participants were (1) a limited ability to provide written informed consent or to understand the questionnaires and tasks, (2) disorders that might influence cognition or motor performance (e.g., craniocerebral injury), (3) magnetic resonance contraindications, (4) current treatment for mental disorders, or (5) use of psychotropic medication or substances. We deliberately excluded illicit substance use, as the resulting neurological damage is rapid (e.g., in methamphetamine use, which is relatively common in the study site region) and their use is much more difficult to objectify (e.g., cannabis and tobacco use often co-occur). Applying the inclusion and exclusion criteria, 855 participants were invited for personal diagnostic screening. In the personal screening, we used the Munich-Composite International Diagnostic Interview (DIA-X/M-CIDI, Wittchen and Pfister 1997) to assess the following exclusion criteria: (6) lifetime psychotic symptoms, bipolar disorder, and other SUD or BA not under study, and (7) major depression, somatoform, anxiety, obsessive compulsive, or eating disorders in the past 4 weeks. At the end of the recruitment phase, suitable individuals were excluded because they were no longer needed for the control group and behavioral addiction group that had already been filled. Finally, 338 participants were included in the study (see Table 1 for participants’ characteristics, Fig. 1 for the participant flow, and the online supplemental materials for the initial sample size calculation). The study protocol was approved by the Institutional Review Board (IRB00001473) at the Technische Universität Dresden (EK45022012).

**Table 1:** Demographic characteristics of the baseline sample with means (*M*) and standard deviations (SD) or numbers (*n*) and percentages for the substance-related disorder (SUD) group, the behavioral addiction (BA) group, and the control group

Baseline sample			
	*SUD*	*BA*	*Control*
*N* = *338*	100	118	120
	*M (SD)*	*M (SD)*	*M (SD)*
Age	21.8 (1.6)	21.8 (1.7)	21.9 (1.8)
Intelligence quotient	103.7 (8.9)	104.4 (10.1)	104.8 (10.4)
	*n* (%)	*n* (%)	*n* (%)
Female participants	53 (53.0%)	70 (59.3%)	76 (63.3%)
Income ≤ 1000 Euro per month	75 (75.8%)	92 (77.0%)	89 (75.4%)
School graduation Gymnasium^a^^, b^	70 (70.7%)	87 (73.7%)	98 (83.0%)
In education, pupils, or students^b^	72 (72.7%)	87 (73.7%)	87 (73.7%)

^a^Gymnasium is a type of secondary schools existing in Germany, which qualifies for university entrance

^b^Three participants had missing values

### Measurements

#### Addictive disorder groups at baseline

At baseline, participants were interviewed with a modified version of the DIA-X/M-CIDI to assess the (adapted) DSM-5 diagnostic criteria for SUD and BA. Out of 100 participants who were included in the SUD group, 45 individuals (45%) met diagnostic criteria for alcohol use disorder, 39 (39%) for tobacco use disorder, and 16 (16%) for both. From 118 participants who were included in the BA group, 83 participants (70%) had a BA related to internet use, 16 (14%) to gaming, 18 (15%) to internet use and gaming, 1 to gambling (1%), and none to shopping. According to the DSM-5 severity specifiers, the severity of SUD (alcohol, tobacco) and BA (internet, gaming, gambling, shopping) in our sample at baseline were mainly mild (62%) and moderate (28%; see Table S2), respectively.

#### Outcome variables: addictive behavior

Consistent with the theoretical assumptions of several addiction models (Griffiths 2005; Shaffer et al. 2004; Vanyukov et al. 2012), we have assumed that many commonalities exist among the various manifestations of addictive behavior (e.g., loss of control over time and quantity) and that these commonalities reflect a shared etiology. To the best of our knowledge, there are no studies demonstrating a specific relationship between EFs and particular addictive behaviors (e.g., alcohol use only).Therefore, we combined all expressions of addictive behavior (i.e., alcohol use, tobacco use, internet use, gaming, gambling, shopping) into the following three outcomes:

1. Quantity of use: The amount of use on a typical occasion was assessed for each of the addictive behaviors (gram ethanol, cigarettes, and hours). The different values for quantity of use were normalized (i.e., rescaled to range between 0 and 1 and to have only positive values for the later addition) in a long data format to make them comparable over the different addictive behaviors and time (baseline and follow-ups), and then summed up to form a composite score at each time point. The sum can range between 0 and 6, according to the rescaling from 0 to 1 and the 6 addictive behaviors.
2. Frequency of use: The frequency of use was assessed as days per week for each addictive behavior. The different frequencies of use were summed up over the different addictive behaviors into a composite score at each time point. The sum can range between 0 and 42, according to the maximum of seven days per week and the 6 addictive behaviors.
3. Addictive disorder severity: The (adapted) DSM-5 criteria were assessed for each addictive behavior. All met addictive disorder criteria were summed up to one score at each time point. The sum can range between 0 and 64, according to the maximum of 11 DSM-5 criteria (with the exception of gambling disorder with 9 criteria) and the 6 addictive disorders.

To examine the construct validity of the composite score of quantity, we used the correlation matrix (Table S3). Since the correlations of quantity of use with frequency of use and the DSM-5 criteria are medium to high (ranging from *r* = 0.40 to *r* = 0.72), we assume a good construct validity of our quantity composite score. In addition, we also distinguished between substance-related and non-substance-related indicators of addictive behavior in our exploratory analyses.

Quantity and frequency of use had continuous data while addictive disorder symptoms were count data. Descriptive statistics for the three addictive behavior outcomes (i.e., quantity and frequency of use and DSM-5 criteria) are shown in Table 2. For detailed information concerning each addictive behavior (i.e., alcohol use, tobacco use, internet use, gaming, gambling, and shopping), see Tables S4-1 to S4-3 in the online supplemental materials. Due to our recruitment strategy (no lifetime BA in the SUD group and no lifetime SUD in the BA group), the substance-related and non-substance-related outcomes are generally low or even negatively correlated (see Table S5). Please note that, although we only have one participant with a gambling disorder and no participant with a shopping disorder at baseline, we still have inter-individual variance in the use and symptoms related to gambling and shopping at baseline and over time.

**Table 2:** Overview of number of participants, time points, and outcomes per assessment year for all participants and separately for each baseline group

		Baseline				Follow-up 1 year^a^				Follow-up 2 years^a^				Follow-up 3 years^a^			
		All	SUD	BA	Control	All	SUD^b^	BA^b^	Control^b^	All	SUD^b^	BA^b^	Control^b^	All	SUD^b^	BA^b^	Control^b^
*n*		**338**	100	118	120	**309**	91	106	112	**195**	64	70	61	**251**	67	89	95
%		**100%**	30%^c^	35%^c^	35%^c^	**91%**^**c**^	30%^c^	34%^c^	36%^c^	**73%**^**c**^	33%^c^	36%^c^	31%^c^	**74%**^**c**^	27%^c^	35%^c^	38%^c^
Time point (years)		**0**	0	0	0	**1.27 (0.21)**	1.28 (0.18)	1.26 (0.20)	1.26 (0.22)	**2.41 (0.19)**	2.34 (0.18)	2.39 (0.19)	2.49 (0.19)	**3.28 (0.22)**	3.30 (0.21)	3.27 (0.27)	3.29 (0.22)
Outcomes																	
Quantity^d^	M (SD)	**0.38** **(0.26)**	0.45 (0.27)	0.46 (0.26)	0.23 (0.18)	**0.60** **(0.35)**	0.77 (0.41)	0.59 (0.31)	0.48 (0.27)	**0.67** **(0.24)**	0.82 (0.31)	0.61 (0.16)	0.56 (0.12)	**0.70** **(0.24)**	0.88 (0.32)	0.67 (0.16)	0.60 (0.17)
	**range**	**0**–**1.6**	.03–1.6	.1–1.2	0– 0.8	**0**–**2.5**	.2–2.5	.2–1.7	.1–1.4	**.2**–**2.6**	.3–2.6	.3–2.0	.2–.9	**0**–**2.5**	.3–2.5	.5–1.4	0–1.3
Frequency^d^	M (SD)	**6.71** **(4.44)**	8.49 (4.90)	8.16 (3.42)	3.81 (3.35)	**9.25** **(4.37)**	11.53 (5.21)	9.52 (3.38)	7.12 (3.35)	**5.51** **(4.11)**	7.95 (4.61)	5.06 (3.63)	3.47 (2.49)	**5.60** **(4.20)**	8.00 (4.67)	5.35 (3.93)	4.13 (3.28)
	**range**	**.3–21.3**	.5–21.3	.8–16	.3–11.5	**.3–23**	1–23	.8–21.5	.3–16.5	**0–20**	.3–20	.5–15.5	0–9.5	**0–20**	1.3–17	0–18.5	0–20
DSM-5 criteria^d^	M (SD)	**3.03** **(2.73)**	4.18 (2.28)	4.61 (2.56)	0.52 (0.63)	**2.10 (2.58)**	2.85 (2.59)	2.70 (3.08)	0.79 (1.22)	**1.48 (2.09)**	2.13 (2.33)	1.87 (2.27)	0.36 (0.78)	**2.12 (2.68)**	2.90 (2.86)	2.73 (3.03)	1.00 (1.62)
	**range**	**0–16**	2–13	2–16	0–2	**0**–**16**	0–11	0–16	0–6	**0**–**11**	0–11	0–10	0–3	**0**–**16**	0–16	0–16	0–8

*SUD*, the substance use disorder group; *BA*, behavioral addiction group

^a^The clinical follow-ups were scheduled according to the date of the baseline fMRI session. This was appropriate for all participants with the exception of six participants, for whom the fMRI session took place after the first baseline clinical assessment with a delay of more than one year. For these six participants, we re-allocated the outcome values to later follow-up waves according to the baseline clinical assessment instead of the baseline fMRI session (e.g., data from a 1-year follow-up that took place 1 year after the participant’s fMRI session, but 2 years after the participant´s baseline assessment, was re-allocated to the 2-year follow-up)

^b^Grouping is based on the baseline group assignments

^c^Refers to % from the baseline sample with the exception of the 2 years follow-up, where for organizational reasons only a sub-sample (*n* = 269) was invited

^d^The possible value ranges for the outcomes were: quantity 0 to 6 (according to the rescaling from 0 to 1 and the 6 addictive behaviors), frequency 0 to 42 (according to the maximum of 7 days per week and the 6 addictive behaviors), and DSM-5 criteria 0 to 64 (according to the maximum of 11 DSM-5 criteria (with the exception of gambling disorder with 9 criteria) and the 6 addictive disorders)

In bold are the values of the complete sample

#### Predictors: executive function tasks

The following nine tasks were used to assess individual differences in EFs and as a basis for modeling latent GEF: Stroop, AX continuous performance, color shape, stop signal, letter memory, number letter, go-nogo, 2-back, category switch. For a detailed description of the tasks, see Table S6 in the online supplemental materials and Wolff et al. (2020; 2016). For 7 out of the 9 tasks, error rates (ERs) and reaction times (RTs) were combined into inverse efficiency scores (IESs; Bruyer and Brysbaert 2011) to account for individual differences in the balance of the speed-accuracy trade-off (Bogacz et al. 2010). ERs included only wrong-key errors. RTs for error trials and trials immediately following wrong-key errors were excluded. RTs below 100 ms and RTs deviating from the median by more than 3.32 median absolute deviations were also excluded (Wilcox and Keselman 2003). IESs were not used for the stop signal task because an adaptive tracking algorithm results in approximately constant ERs. The stop signal task was analyzed according to the quantile method proposed by Congdon et al. (2012). IESs were also not used for the letter memory task, where speeded responses were not required. ERs of the letter memory task were arcsine-transformed to improve normality. Table 3 provides descriptive statistics and reliabilities of the EF tasks (for more information, see Wolff et al. 2020; for descriptive statistics per group see Table S7 in the supplemental material).

**Table 3:** Descriptive statistics and reliabilities of the executive function tasks

Task	*M* (*SD*)	Range	Skewness	Kurtosis	Reliability^a^
Go-nogo	394 ms (86)	281–671	1.47	2.13	0.81
Stop signal	182 ms (50)	61–336	0.07	− 0.22	0.59
Stroop^b^	63 ms (37)	0–177	0.78	0.70	0.53
Number-letter^b^	332 ms (203)	0–963	1.11	1.24	0.82
Color-shape^b^	109 ms (82)	0–371	1.13	1.21	0.64
Category switch^b^	81 ms (87)	0–355	1.40	1.52	0.68
2-back	549 ms (172)	288–1089	0.99	0.74	0.88
Letter memory^c^	0.43 (0.22)	0–1.08	− 0.18	0.13	0.61
AX-CP^d^	368 ms (55)	237–534	0.60	0.50	0.69

^a^Internal consistency was calculated by adjusting split-half correlations with the Spearman-Brown prophecy formula

^b^Difference scores (Stroop interference and switch cost, respectively) were replaced with 0 when negative

^c^Accuracy scores were arcsine-transformed to improve normality

^d^Inverse efficiency scores (IESs) could not be calculated for 2 participants who had no correct BX trials. These observations were assigned the maximum (534 ms)

#### Control variables

Due to the non-experimental nature of observational studies, considering control variables (i.e., variables that influence addictive behavior, but are not in the focus of the study) is important to facilitate causal interpretations. Based on the existing literature on addictive behavior and their development over time in young adults (e.g., Mortensen et al. 2006; Sjölund et al. 2015; Wittchen et al. 2008b), we predicted that higher age, male gender, lower intelligence, and lower education level would be related to higher initial values and a stronger increase of addictive behavior over time. According to our hypotheses, we held these control variables constant in our longitudinal analyses to better understand the relationship between GEF and addictive behavior. Moreover, the group allocation at baseline would per definition be related to use and addictive disorder severity and therefore was included as a control variable. Group allocation, age, gender, and school graduation were measured at the first personal appointment using a modified version of the DIA-X/M-CIDI (Wittchen and Pfister 1997). Intelligence quotients (IQ) were assessed at the second appointment using the Hamburg-Wechsler Adult Intelligence Scale-Revised, (HAWIE-R; Tewes 1994), a German version of the Wechsler Intelligence Test (WAIS). Based on a reviewer’s comment, we created a structural equation model (SEM) to test the relationship between IQ and GEF. We found a significant positive relationship (*β* = 0.38, 95%CI 0.18–0.58, *p* < 0.001), which is comparable to the results of Friedman and Miyake (2017) on the correlation of GEF with the general *g* factor (*r* = 0.5). We included IQ as a covariate in the analysis because we believe that the GEF factor reliably predicts real-world behavior even when holding intelligence constant (Friedman and Miyake 2017; Friedman and Robbins 2022). We also presented our analysis without covariates to avoid potential bias in our results due to covariates.

### Dropout analyses

We performed a group comparison between the participants who dropped out at some point during the 3 years (26%) and those who did not (Table S8 in the online supplemental materials). These analyses showed that there were no significant differences between the drop-outs and the completers, except for IQ, which was lower in the drop-outs (*M* = 102.53, SD = 1.01) compared to completers (*M* = 105.02, SD = 0.62; *t* =  − 2.03, *p* = 0.04). Since we controlled for IQ in our analyses, we consider that there is no systematic attrition bias in our study findings.

### Data analyses

To test Hypothesis 1, we used our previous SEM model of GEF (Wolff et al. 2020), described in the next subsection, and compared the latent factor GEF from this model between our three baseline groups using the measurement invariance approach. To test the Hypotheses 2 and 3, we specified latent growth models for each outcome variable (quantity, frequency, DSM-5 criteria) and tested whether the latent factor GEF is associated with the intercept (cross-sectional relationship) and slope (longitudinal relationship) of each of the addictive behavior outcomes. A detailed description of the analysis steps used to test each hypothesis is provided in the flowing two subsections. Mplus 8.4 (Muthén and Muthén 1998–2017) was used for data analysis. Preliminary analyses showed that no specific variable was significantly associated with missingness. Data were analyzed using robust maximum likelihood (MLR) estimation to account for the skewed data (Table S9 in the supplemental material) and full information maximum likelihood (FIML) to handle missing data. Our hypotheses have not been pre-registered before the data collection. For research transparency, the project protocol, the primary data, and the Mplus files were uploaded on the Open Science Framework (OSF) under https://osf.io/yu5rm/ and under https://osf.io/zdqve/, respectively.

### Hypothesis 1: Group differences in executive functioning at baseline

#### Basic model of general executive functioning

**Fig. 2:**
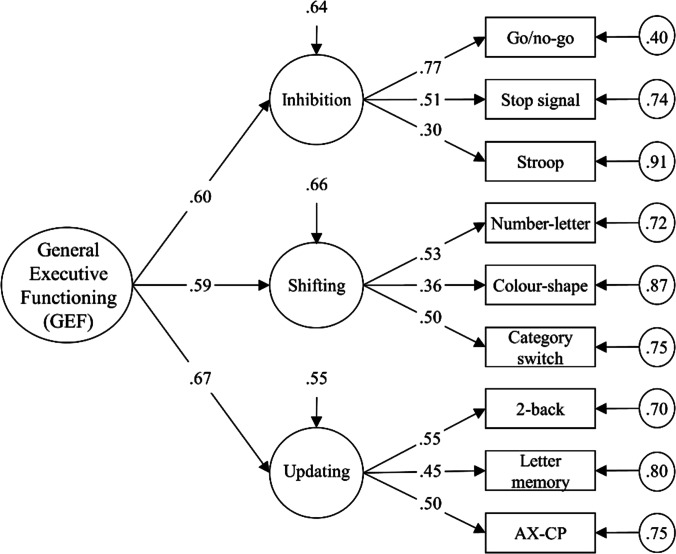
Second-order-factor measurement model of latent executive functioning which served as the basis for all of the analyses (Wolff et al. 2020). Standardized values are reported. All estimates were significantly different from zero (*p* < .001)

Previous work (Wolff et al. 2020) combined the nine EF tasks from our project following the approach introduced by Miyake et al. (Miyake et al. 2000) in which three first-order factors and a common second-order factor were derived using latent variable analyses. The measurement model included the three correlated first-order factors inhibition, shifting, and updating, each representing abilities in one EF as indicated by three respective task outcomes, and the second-order factor GEF (Fig. 2). The model was compared against alternative models and selected applying Karr et al.’s (2018) lenient criteria for model acceptance (see Wolff et al. 2020 for additional information). This measurement model showed acceptable fit to the data with a confirmatory fit index (CFI) of 0.93 and a root mean square error of approximation (RMSEA) of 0.04.

#### Test of group differences in general executive functioning

To test the hypothesis that addictive disorders are characterized by lower GEF (Hypothesis 1), we compared the three groups on the GEF factor. As a prerequisite for meaningful group comparisons, we tested for measurement invariance of our second-order factor model following the steps recommended by Rudnev et al. (2018). Tests of configural, metric, and scalar invariance of first- and second-order models across the groups were conducted within the framework of multiple-group confirmatory factor analysis. As the first step, a baseline (non-invariant) model with no constraints (representing configural invariance) was fitted to the data. The baseline model with no equality constraint was simultaneously tested across the three groups. As can be seen in Table 2 (M1^1^), the fit of this model to the data was acceptable, indicating that configural invariance was supported. Model fit was evaluated using χ^2^, CFI, the Tucker-Lewis index (TLI), standardized root mean square residual (SRMR), and RMSEA (Putnick and Bornstein 2016). Equality constraints were then imposed on first-order (M21) and second-order factor loadings (M3) across the three groups. If the increase in model fit to a more constrained model was non-significant, metric and/or scalar invariance was assumed. Based on research by Chen (2007), acceptable model fit for more restrictive invariant models are as follows: a ΔCFI of >  − 0.01, supplemented by a ΔRMSEA of < 0.015 or a ΔSRMR of < 0.03 for tests of factor loading invariance; a ΔCFI of >  − 0.01, supplemented by a ΔRMSEA of < 0.015 or a ΔSRMR of < 0.01 for tests of intercept invariance. A non-significant change of *χ*^2^, ΔCFI, ΔRMSEA, and ΔSRMR from model M1 to the more constrained model M2 and from M2 to M3 indicated full metric invariance (Table 4). Next, equality constraints were imposed on all item intercepts of the first-order (M4) and the second-order factors (M5) to test scalar invariance. A non-significant change of *χ*^2^, ΔCFI, ΔRMSEA, and ΔSRMR from model M3 to the more constrained model M4 and from M4 to M5 indicated scalar invariance. This conclusion is further supported by the decreasing fit indices AIC and BIC from M1 to M5. These results supported the measurement invariance across our three groups and support the assumption that our subsequent group comparisons of the latent means are valid. Given that the first- and the second-order factors were scalar invariant, we proceeded with comparing latent means of the GEF factor across the groups, using the parameters of the last model (M5). In this model, the latent factor means in one group were constrained to zero, whereas the latent means in the other two groups were freely estimated.

**Table 4:** Results of measurement invariance tests of the second-order factor model of executive functioning

	*χ*^2^	Δ*χ*^2^	df	CFI	ΔCFI	RMSEA	ΔRMSEA	SRMR	BIC	AIC
M1. Configural	93.52	-	73	0.90	-	0.05	-	0.07	8477.78	8419.85
M2. First-order metric	102.41	8.89	85	0.92	0.02	0.04	0.01	0.07	8454.85	8404.73
M3. First- and second-order metric	103.75	1.34	88	0.93	0.01	0.04	0.00	0.07	8448.24	8400.07
M4. First-order scalar	103.90	0.14	103	0.99	0.07	0.01	0.03	0.07	8408.62	8370.22
M5. First- and second-order scalar	103.81	0.09	104	1.00	0.004	0	0.01	0.07	8405.88	8368.13

*df*, degrees of freedom; *CFI*, comparative fit index; *RMSEA*, root mean squared error of approximation; *SRMR*, standardized root mean square residual; *BIC*, sample size adjusted Bayesian information criterion; *M1*, baseline (noninvariant) model with no constraints (representing configural invariance); *M2–M5*, models with step-wise constraining of factor loadings (metric invariance) and intercepts (scalar invariance) for the first- and second-order factors across groups to test metric and scalar invariance

### Hypotheses 2 and 3: Associations between executive functioning and addictive behavior

#### Latent growth curve models

**Fig. 3:**
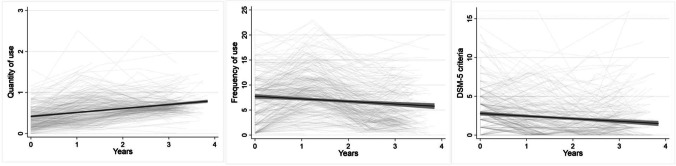
Individual trajectories (light gray) and mean trajectory (black) with 95% confidence interval (dark gray) of the outcomes quantity of use, frequency of use, and number of met DSM-5 criteria for addictive disorders from baseline (year 0) to the 3 years follow-up used for the latent growth modelling

**Fig. 4:**
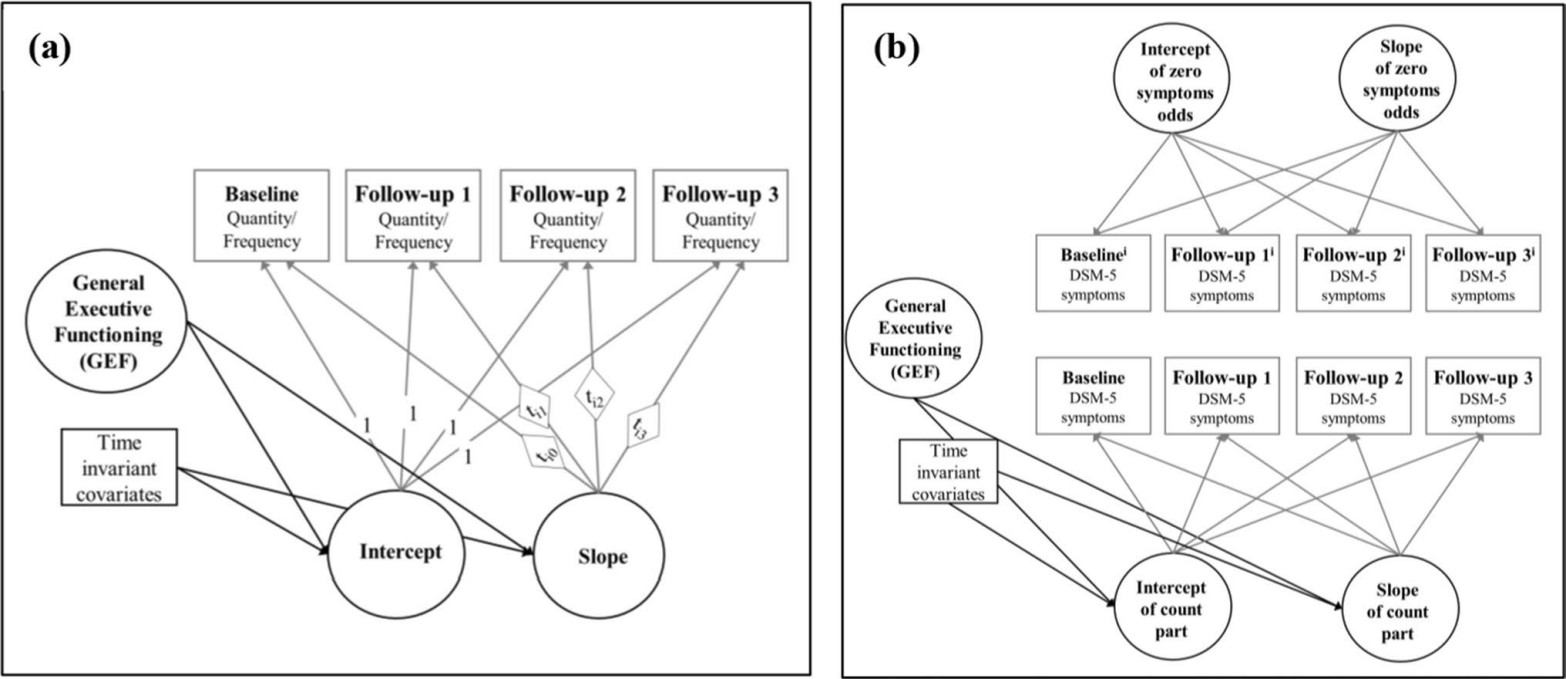
The conditional latent growth models for the trajectories of (**a**) the quantity and frequency of use and (**b**) the DSM-5 symptoms, both with the predictor latent general executive functioning (GEF) and the time invariant control variables age, gender, IQ, school graduation, and group membership at baseline. Note. ^i^first class of the Zero-inflated Poisson model for DSM-5 symptoms refers to the zero-inflation part with only values of zero in all measurements

We hypothesized that lower GEF is related to more addictive behavior at baseline (Hypothesis 2) and to an increase in addictive behavior over time (Hypothesis 3). These hypotheses were tested with latent growth curve models (LGMs). The individual intercepts and trajectories of the addictive behavior outcomes are depicted in Fig. 3. The LGM approach allows for the estimation of the means and variances of latent intercept and slope factors from these individual trajectories. The intercept refers to the mean baseline levels of addictive behavior, whereas the slope represents the mean trajectories of addictive behavior over time. Data of addictive behavior were right-skewed, i.e., the sample was more likely to report smaller rather than larger values in the quantity and frequency of use and the number of addictive disorder criteria (see Table 2 and Tables S4-1 to S4-3 in the online supplemental materials). This is in line with previous data and can be justified given our recruitment strategy in a non-clinical population. For quantity and frequency of use, data were continuous. We used MLR to obtain estimations that are robust to non-normality. Because it is important to take the variance in the assessment time points within each wave into account (see Table 2; Aydin et al. 2014), we used the multilevel modeling (MLM) approach in Mplus, which allows time to be a variable with a random slope (reflecting individually varying times of observations). Two linear LGMs allowing for individually varying time scores (option TSCORES) were conducted, one for the quantity and one for the frequency of use over the three follow-ups (Fig. 4a). Table 5 shows the estimated parameters of the LGMs for each outcome variable. For the quantity and frequency of use, the intercept and the slope differed significantly from zero. The slope of quantity increased over time while the slopes of frequency decreased. There were significant within-group variations in intercepts and slopes, which means that there is significant variability in the initial values and growth rates.

**Table 5:** Results of the latent growth models for each outcome variable

		Intercept mean	Intercept variance	Slope mean	Slope variance	Intercept with Slope
Quantity of use		0.38*	0.07*	0.14*	0.01*	− 0.02*
Frequency of use		7.30*	11.22*	− 0.48*	0.74*	− 0.79
DSM-5 criteria	First class of the ZIP model (log odds of inflated zero)	0.00^a^	3.20	0.09	0.13	0.26
	Second class of the ZIP model (log count from Poisson part)	1.00*	0.32*	− 0.23*	0.05*	0.03

Unstandardized values are reported. The possible value ranges for the outcomes were: quantity 0 to 6 (according to the rescaling from 0 to 1 and the 6 addictive behaviors), frequency 0 to 42 (according to the maximum of seven days per week and the 6 addictive behaviors), and DSM-5 criteria 0 to 64 (according to the maximum of 11 DSM-5 criteria (with the exception of gambling disorder with 9 criteria) and the 6 addictive disorders). The observed value ranges were quantity 0 to 2.6, frequency 0 to 23, and DSM-5 criteria 0 to 16 (see Table 2)

*ZIP*, zero-inflated Poisson

^a^Parameter fixed at 0

^*^Bold indicates significance at *p* ≤ 0.05

DSM-5 criteria (addictive disorder severity) were count data with a zero-inflated distribution. Therefore, we conducted LGMs for count outcomes using a zero-inflated Poisson (ZIP) model (Fig. 4b). The ZIP model simultaneously estimates a binary model (i.e., the probability of developing one or more symptoms over time) and a count trajectory (i.e., the number of DSM-5 symptoms among those who report symptoms) (e.g., Liu and Powers 2007). Our final ZIP model does not involve individually varying time points because convergence issues were encountered when estimating change models with count outcomes (Grimm and Stegmann 2019). For the DSM-5 symptoms, the odds of an inflated zero (no symptoms) increased non-significantly while the expected Poisson counts decreased significantly over 3 years (Table 5). In other words, there is a very low probability that participants without symptoms develop symptoms during the study period. In participants with one or more symptoms, we found evidence that they experienced a decline in the symptoms. The directions of the trajectories do slightly differ between the baseline groups (Fig. S1 in the online supplemental materials), which further justifies the use of the baseline group as a control variable if one is interested in the general associations between GEF and addictive behavior trajectories. There were significant within-group variations in intercept and slope of the poison part of the ZIP model while it was non-significant in the zero-inflated part. This means that all participants had a comparable probability to stay in the group without symptoms.

#### Test of cross-sectional and longitudinal relationships

To test our hypotheses, we included the predictor GEF in the LGMs and examined the association between GEF and the intercept (for Hypothesis 2) and the slope (for Hypothesis 3). For quantity and frequency, GEF was the predictor of latent intercepts and slopes (Fig. 4a). For DSM-5 criteria, the individually varying trajectories for the expected Poisson counts of the ZIP model were of interest (i.e., predictor of the change in the symptoms; Fig. 1b). Therefore, we fixed the variance for the individual trajectories of change in the odds at zero. Based on theoretical assumptions and empirical evidence about control variables of the relationship between EFs and addictive behavior (see subsection “Control variables”), these LGMs were adjusted for the time invariant covariates group membership, age, gender, IQ, and school graduation at baseline (see subsection “Control variables”). The unadjusted results are presented in Table S10 in the supplemental materials.

#### Exploratory analyses

As it is important to understand common underlying mechanisms of substance-related and non-substance-related addictive behavior, we conducted additional exploratory LGMs that also included GEF as predictor and the same three outcome measures as the LGMs for testing our hypotheses, but separately for the substance-related and non-substance-related addictive behavior outcomes, resulting in six additional exploratory LGMs. Upon request during the review process, we performed supplementary LGMs separately for each baseline group, including the predictor GEF. These exploratory analyses modeled the course of substance-related addictive behaviors in the SUD group, non-substance-related addictive behaviors in the BA group, and overall addictive behaviors in the control group.

## Results

### Hypothesis 1: Group differences in general executive functioning at baseline

We found no evidence for Hypothesis 1 that GEF is lower in the addiction groups compared to the control group at baseline. The mean group differences were zero (SUD vs. control: 0.01; BA vs. control: 0.01; SUD vs. BA: 0.00) and the 95% confidence intervals were also narrow around zero (SUD vs. control: − 0.18 to 0.19; BA vs. control: − 0.19 to 0.18; SUD vs. BA: − 0.19 to 0.19), which indicated that there was only a very low probability for group difference.

### Hypothesis 2: Cross-sectional associations between executive functioning and addictive behavior

#### Quantity and frequency of use

The predictors and outcomes were standardized before the analyses to yield estimates that have the same range as correlation coefficients. Contrary to Hypothesis 2, the conditional LGM revealed a moderate association of 0.33 between higher GEF and higher quantity of use at baseline in the adjusted model (Table 6). The unadjusted model yielded comparable estimates (Table S10 in the supplemental materials). The relationship between GEF and the frequency of use at baseline was non-significant (95% CI − 0.06 to 0.61).

**Table 6:** Results of the adjusted^a^ conditional latent growth models testing the relationship between the quantity of use, frequency of use, and DSM-5 criteria for addictive disorders over time and the predictor general executive functioning (GEF)

			Estimates^b^	*p*-values	95% confidence intervals
Quantity of use		Intercept	0.33	0.04	0.001–0.66
		Slope	− 0.12	0.04	− 0.25 to -0.001
Frequency of use		Intercept	0.24	0.09	− 0.04 to 0.54
		Slope	− 0.13	0.04	− 0.27 to -0.001
DSM-5 addictive disorder criteria	Second class of the ZIP model (log count from Poisson part)	Intercept	0.06	0.32	− 0.10 to 0.23
		Slope	− 0.17	0.31	− 0.60 to 0.26

ZIP, zero-inflated Poisson

^a^Relationship adjusted for the time invariant control variables baseline group membership and demographic characteristics (age, gender, IQ, and school graduation). Unadjusted results are displayed in Table S10 of the supplementary material

^b^Standardized estimates

Results of the exploratory LGMs showed moderate positive associations between higher GEF and higher quantity and frequency of non-substance-related use at baseline (95% CI quantity: 0.05–0.66; 95% CI frequency: 0.00–0.59). There was no evidence for such associations with substance-related use at baseline (95% CI quantity: − 0.25 to 0.38; 95% CI frequency: − 0.31 to 0.30, see Tables S11-1 and S11-2 in the online supplemental materials). The 95% confidence intervals of the supplementary groupwise LGMs showed that there were comparable associations between GEF and the quantity and frequency of use over all three groups (Tables S12-1 and S12-2). These associations were more likely to be positive, i.e., a higher GEF predicts more use at baseline.

In sum, there was no evidence for a cross-sectional negative relationship between lower GEF and higher frequency of use. However, we found evidence for a moderate association between higher GEF and lower quantity of use at baseline, which was probably due more to non-substance-related behaviors (internet use, gaming, gambling, shopping) than to substance-related use (smoking, alcohol use). The supplementary groupwise results also provided evidence of a positive relationship between GEF and addictive behavior, but no evidence for differences among the three baseline groups.

### Outcome: DSM-5 addictive disorder criteria

For the DSM-5 criteria, the adjusted ZIP model showed no significant association between lower GEF and more addictive disorder criteria at baseline (95% CI − 0.10 to 0.23; Table 6). The unadjusted model also revealed no evidence of an association (Table S10 in the online supplemental materials).

The exploratory analyses revealed comparable 95% CIs of the relationships between baseline GEF and substance-related disorder criteria versus behavior addiction criteria (Table S11-3 in the online supplemental materials). The 95% confidence intervals of the supplementary groupwise LGMs showed that there were comparable associations between GEF and the course of DSM-5 criteria over all three groups (Table S12-3). These associations were predominantly positive, i.e., a higher GEF was associated with more DSM-5 criteria at baseline.

### Hypothesis 3: Longitudinal associations between executive functioning and addictive behavior

#### Quantity and frequency of use

As predicted by Hypothesis 3, results revealed significant associations between lower GEF and a higher increase in the quantity (− 0.12) as well as a smaller decrease in the frequency of use (− 0.13) over time (Table 6). The confidence intervals of the estimates in the unadjusted models were comparable (Table S10 in the supplemental materials). Scatterplots of the negative relationships between the factor scores of latent GEF and the latent slopes are displayed in Fig. S2 in the online supplemental materials.

The 95% confidence intervals of the estimates from the exploratory LGMs showed that these longitudinal associations were quite comparable between substance-related and non-substance-related addictive behavior (Tables S11-1 and S11-2 in the supplemental materials). The supplementary groupwise LGMs also supported our results from the whole-sample analyses. The 95% confidence intervals of the estimates from these LGMs consistently showed that the relationship between GEF and quantity and frequency of use over time tended to be negative in all three groups, i.e., a higher GEF is associated with greater increases (quantity) and a smaller decreases (frequency) in use over time (Tables S12-1 and S12-2).

### Outcome: DSM-5 criteria

The adjusted and unadjusted models consistently revealed no evidence of a negative association between lower GEF and an increased number of DSM-5 criteria over time. The association was more likely to be negative (− 0.17, 95% CI − 0.60 to 0.26), but it was non-significant (Table 6).

The exploratory analyses revealed comparable 95% CIs of the estimates for the relationships between substance-related versus behavior addiction criteria (Table S11-3 in the supplemental materials). The supplementary groupwise LGMs revealed that the association between GEF and DSM-5 criteria are comparable in all three groups, in that they tend to be negative across the models (Tables S12-3).

## Discussion

Our overall aim was to test the cross-sectional and longitudinal relationships between lower GEF and addictive behavior, going beyond previous studies by operationalizing GEF as a latent variable capturing the shared variance in multiple tasks and predicting separate trajectories for use and symptoms of addictive disorders in young adults. We applied a longitudinal design over three years with a community sample. Our results revealed (1) no evidence for lower GEF in addictive disorders compared to controls, (2) no evidence for a relationship between higher GEF and more addictive behavior at baseline (expect for non-substance-related quantity of use), and (3) evidence that higher GEF is related to more quantity and frequency of use over time. These new findings suggest that lower EFs result in a loss of control over use over time and that addictive disorders may develop after a prolonged period of addictive use or secondarily from interaction with other vulnerability factors. This implies that previous etiological models that assume lower EF as a direct vulnerability factor for addictive disorders need to be refined.

In young adults from a community sample, we found no evidence that individuals with addictive disorders display lower latent GEF compared to healthy controls (Hypothesis 1). These results are in line with a previous study applying a latent variable approach (Gustavson et al. 2017). The authors concluded that EFs are not related to addictive disorders but to other aspects of substance use such as the number of substances ever used. Our current findings seem not to contradict the substantial body of research demonstrating poor EFs in addictive disorders (for meta-analyses, see Chowdhury et al. 2017; Smith et al. 2014; Stephan et al. 2017). Yet, we believe that some of the emerging differences can be explained considering the methodological differences between our study and previous ones. Most of these previous studies were based on case–control designs with patient samples and compared the performance between addictive disorder patients and healthy controls in complex EF tasks. Compared to such patient samples, our sample was relatively young, randomly recruited from the community, and displays a mild to moderate addictive disorder severity. Addictive disorder patients with a longer history of chronic substance use and a higher addictive disorder severity may display lower EF abilities, presumably as a consequence of the addictive disorder (De Wit 2009) or comorbid mental disorders such as depressive or anxiety disorders (Castaneda et al. 2008). Moreover, addictive disorder patients differ in other aspects that are important for EF task performance such as attention deficits due to craving (Naim-Feil et al. 2013) or lower performance motivation (Scheurich et al. 2004). These aspects were less represented in our purer measures of EFs achieved with latent variable modeling.

Concerning the course of addictive behaviors over the study period, we found that participants consumed a higher quantity per occasion, consumed less frequently, and experienced fewer addictive symptoms over time. In line with the results for the group comparisons at baseline, our results showed weak to no evidence that lower general EF abilities are related to a higher frequency of use and a higher number of addictive symptoms at baseline (Hypothesis 2). Interestingly, there was evidence for a moderate positive association between higher GEF and higher quantity of use at baseline, which was against our hypothesis. Exploratory analyses revealed that this positive relationship was mainly evident for addictive behavior without substance use. Higher GEF in our young sample was related to more non-substance-related use (internet use, gaming, gambling, and shopping). One explanation could be that we have included non-pathological and pathological use in our quantity measure. It has been shown that one cannot separate engaged gaming from gaming disorder by looking only at the amount or frequency of use (Peeters et al. 2019). For example, it could be possible that higher GEF is associated with increased but healthier use of the Internet, such as information seeking. Against this explanation, our supplementary groupwise analyses revealed 95% confidence intervals that were more positive. In other words, higher GEF was more likely to be associated with higher substance-related and non-substance-related use at baseline in the SUD and BA groups, respectively. However, these preliminary results should be interpreted with caution because these were exploratory analyses with small sample sizes that yielded very wide confidence intervals. Further studies are needed that better distinguish between healthy and pathological use.

Finally, we found that lower GEF predicted a stronger increase in quantity and a smaller decrease in the frequency of use over time (Hypothesis 3). Exploratory LGMs showed that the 95% CIs of these associations were comparable between substance- and non-substance-related behaviors. Furthermore, the groupwise supplementary analyses showed that these results were comparable over all three baseline groups (SUD, BA, and control group). However, there was no evidence for an association between lower GEF and the trajectories of addictive disorder severity. One explanation could be that we underestimated the true effects in our young community sample with no to low addictive disorder severity. In future analyses, we want to explore whether there are latent groups based on the addictive behavior trajectories and whether these groups differ in baseline GEF (see section II of our registration on https://osf.io/yu5rm/). Another explanation could be that impaired GEF may play a more important role in the development of use than in the development of addictive disorder symptoms. This would be in line with the assumption that EFs are related to a general tendency to engage in externalizing behaviors, rather than a specific vulnerability factor for addictive disorders (Gustavson et al. 2017). This provides new insight into the role of EFs in the etiology and course of addictive behavior. The small size of the predictive associations between GEF and the course of use is in line with previous findings (e.g., Fernie et al. 2013; Gustavson et al. 2017; Khurana et al. 2013; Nigg et al. 2006) and underlines that lower EFs have to be considered in interaction with other factors. As one example, previous findings from the same study showed that “hot” cognitive control in tasks on impulsive decision-making is more related to addictive disorder severity than to use after 1 year (Kräplin et al. 2020). Applying the terminology of Friedman and Robbins (2022), one could conclude that “cold” cognitive control may be important for initial use and the escalation of use, while “hot” cognitive control may be more important for the development of addictive disorder symptoms, such as the devaluation of the negative consequences of the use (MacKillop et al. 2011).

One strength of the study is the recruitment of a representative community sample of adults aged 19 to 27. The young age cohort allows analyses of very early developmental processes of addictive behavior. However, this recruitment may have led to a sampling bias. Respondents were more likely to be younger and female compared to non-respondents. As intended with our design, we also have a high baseline proportion of individuals with mild and moderate addictive disorder severity in the addiction groups. In addition, at the time of designing our original study, there were no established criteria for the diagnosis of BA (except for gambling disorder). To achieve a homogenous group definition, BA was diagnosed using modified DSM-5 SUD criteria that had a lower threshold (two or more criteria) than for the diagnosis of gambling disorder (four or more criteria) or internet gaming disorder suggested in the DSM-5 (five or more criteria). A sample with all these characteristics (younger, more females, lower addictive disorder severity, and higher executive functioning) would lead to an underestimation of true associations. Another strength of the study is the high internal validity of our cross-sectional group comparison at baseline. We only allocated participants to the legal substance use disorder group or the behavioral addiction group who did not meet the criteria for the other addiction group during their lifetime. The disadvantage of this approach is that we had to exclude many respondents who met symptoms of both legal substance use disorders and behavioral addictions during their life. Furthermore, we excluded use of illicit substance at baseline. Both exclusion criteria could contribute to less severe cases in our young sample (e.g., high-risk individuals would be more likely to use illicit substances), and could lead to an underestimation of true effects and to a lower external validity of our results. For example, we cannot generalize our results to people with comorbid substance- and non-substance-related disorders or with illicit substance use (disorders). Another limitation that needs to be discussed is that we used an overall measure of quantity, frequency, and symptoms across six addictive behaviors (or separately for substance-related and non-substance-related addictive behaviors in our explorative analyses). If only one of the six addictive behaviors had been included, we would not have been able to directly test whether associations with EFs differed across the six addictive behaviors because of the small sample size. With this study, we were able to show that these associations are present when all addictive behaviors are combined. Valuable studies focusing specifically on one substance or addictive behavior, such as alcohol use (Heinz et al. 2020) or online gaming (Brand et al. 2021), are underway and will help to understand commonalities and differences in the role of EFs in specific addictive behaviors.

This is the first study to examine the relationships between latent EFs and latent individual trajectories of addictive behavior, including quantity and frequency of use and number of DSM-5 diagnostic criteria met across different forms of addictive behavior (e.g., alcohol or internet use). Results indicate that lower GEF is predictive of the development of use rather than the development of addictive disorder symptoms in young adults with mainly no or low addictive disorder severity. Our findings have important methodological and theoretical implications for future research. Concerning the methods, large representative population studies with adolescents and young adults and sufficiently long study periods are needed to better understand the etiological vulnerability factors of addiction. The predominate use of patient samples can be misleading in that we have previously concluded early vulnerability factors based on evidence from very advanced stages of addiction development. Concerning the theory, our evidence implies that previous etiological models that assume lower EF as a direct vulnerability factor for addictive disorders need to be refined. Addiction models should incorporate the idea that EFs are more closely related to initial use and progression of use, and that addictive disorders develop after a prolonged period of addictive use or secondarily, probably in interaction with other vulnerability factors.

## Supplementary Information

Below is the link to the electronic supplementary material.

Supplementary file1 (DOCX 1.13 MB)

## Footnotes

1 For model identification of the models M1 and M2, we fixed the residual variance of the first-order factor updating at zero in the SUD group.
