# Supplementary material for: The relationship between executive functioning and addictive behavior: new insights from a longitudinal community study
Source: Psychopharmacology (Berl). 2022 Oct 3;239(11):3507–24. doi: 10.1007/s00213-022-06224-3 (PMC9584881; doi:10.1007/s00213-022-06224-3)
Supplement: Supplementary file 1 — Supplementary file1 (DOCX 1.13 MB) [file 213_2022_6224_MOESM1_ESM.docx]

**Supporting information for**

**The relationship between executive functioning and addictive behavior: New insights from a longitudinal community study**

Anja Kräplin^1^, Mohsen Joshanloo^2^, Max Wolff^1,3,4,5^, Klaus-Martin Krönke^1^, Thomas Goschke^1^, Gerhard Bühringer^1,6,7^, Michael N. Smolka^3^

^1^ Faculty of Psychology, Technische Universität Dresden, Germany

^2^ Department of Psychology, Keimyung University, Daegu, South Korea

^3^ Department of Psychiatry, Technische Universität Dresden, Germany

^4^ MIND Foundation, Berlin, Germany

^5^ Charité – Universitätsmedizin Berlin, corporate member of Freie Universität Berlin and Humboldt-Universität zu Berlin, Department of Psychiatry and Psychotherapy, Campus Charité Mitte, Berlin, Germany

^6^ IFT Institut für Therapieforschung, Munich, Germany

^7^ Department of Clinical Research, Faculty of Health, University of Southern Denmark

Correspondence address: Dr. rer. nat. habil. Anja Kräplin, Work group Addictive Behaviors, Risk Analysis and Risk Management, Faculty of Psychology, Technische Universität Dresden, Chemnitzer Straße 46, D-01187 Dresden, Germany. Email: anja.kraeplin@tu-dresden.de

# Sample size calculation

The sample size for the whole project was estimated using Stata 13 for multiple linear regression with power (1- β) = .80, significance level α= 0.05, and five covariates (age, gender, IQ, income, school graduation). We assumed moderate group differences at baseline with R^2^=.05 according previous studies comparing individuals with gambling disorder, alcohol dependence, and Tourette syndrome on measures of executive functions and decision-making (Goudriaan et al. 2005; Goudriaan et al. 2006). The necessary sample size would have been N=235 in total. Furthermore, we assumed a drop-out rate of 30% during the first funding phase of the project (3 years). The final estimated sample size was 330 with 110 in each group.

Please note that the sample size calculation was not conducted for the presented data analyses with latent general executive functioning (GEF) as predictor in latent growth models (LGMs). Based on our relatively large sample size at baseline (n=338), we decided to apply a latent variable approach to operationalize executive function abilities to account for recent research (Gustavson et al. 2017). We found an acceptable model fit in a previous study (Wolff et al. 2020), which we present in more detail in the subsection ‘Basic model of executive functioning’ of the Methods section. After successful latent modelling of GEF, we decided to use GEF as predictor in a LGMs. Concerning LGMs, Fan and Fan (2005) showed with Monte Carlo simulations that LGMs advantageous over other statistical approaches (i.e., repeated measures univariate ANOVA, multivariate ANOVA, and t-tests) in terms of power for detecting moderate growth under small sample size conditions. Moreover, missing data in LGMs can be handled by FIML estimation. The successful convergence of our models and the model characteristics convinced us that we can apply this approach to find reliable and valid results.

# Respondents and non-respondents

A random sample of 18.000 inhabitants between 19 and 27 was taken and from the registration office files of Dresden, Germany, from 2013 to 2016 and invited by post to participate in our study. 1,856 inhabitants responded to our invitation letter (10.3 %). We descriptively compared respondents and non-respondents regarding their birth year and gender. Respondents were more likely to be from the birth cohorts 1990, 1991, 1992, 1993, 1994, and 1996 and more likely to be female (Table S1).

# Table S1 Gender and birth years of respondents and non-respondents to the postal study invitation.

|  | Respondents | Non-respondents | Test statistics |
| --- | --- | --- | --- |
|  | n (%) | n (%) |  |
| Total | 1,856 | 16,144 |  |
| Gender |  |  |  |
| Female | 1,077 (58.03%) | 7,760 (48.07%) | χ2=66.04, *p*<.001 |
|  |  |  |  |
| Birth year |  |  | Overall: χ2=79.15, *p*<.001 |
| 1988 | 82 (4.4%) | 668 (4.1%) | χ2=.33, *p*=.57 |
| 1989 | 70 (3.8%) | 680 (4.2%) | χ2=.81, *p*=.37 |
| 1990 | 58 (3.1%) | 692 (4.3%) | χ2=5.63, *p*=.02 |
| 1991 | 55 (3.0%) | 695 (4.3%) | χ2=7.51, *p*=.01 |
| 1992 | 362 (19.5%) | 2.638 (16.3%) | χ2=11.99, *p*=.001 |
| 1993 | 360 (19.4%) | 2.640 (16.4%) | χ2=11.10, *p*=.001 |
| 1994 | 347 (18.7%) | 2.652 (16.4%) | χ2=6.17, *p*=.01 |
| 1995 | 319 (17.2%) | 2.681 (16.6%) | χ2=.40, *p*=.53 |
| 1996 | 203 (10.9%) | 2.797 (17.3%) | χ2=48.92, *p*<.001 |

# **Table S2** Severity of substance-related and additive disorders at baseline according to the DSM-5 specifiers separately for the substance use disorder (SUD) group, the behavioral addiction (BA) group, and the control group.

|  | **SUD**  **n=100** | **BA**  **n=118** | **Controls**  **n=120** |
| --- | --- | --- | --- |
| DSM-5 SUD  Tobacco-related  Mild  Moderate  Severe  Alcohol-related  Mild  Moderate  Severe | n=61  53%  33%  14%  n=55  84%  11%  5% | n=0  n=0 | n=0  n=0 |
| (Adapted) DSM-5 BA  Internet-related  Mild  Moderate  Severe  Gaming-related  Mild  Moderate  Severe  Gambling-related  Mild  Moderate  Severe  Shopping-related  Mild  Moderate  Severe | n=0  n=0  n=0  n=0 | n= 101  61%  31%  8%  n=34  44%  32%  24%  n=1  100%  0%  0%  n=0 | n=0  n=0  n=0  n=0 |

# Table S3 Correlation between the addictive behavior outcomes according to the assessment years

|  | **Baseline** | | **Follow-up 1 year** | | **Follow-up 2 years** | | **Follow-up 3 years** | |
| --- | --- | --- | --- | --- | --- | --- | --- | --- |
|  | **Quantity** | **Frequency** | **Quantity** | **Frequency** | **Quantity** | **Frequency** | **Quantity** | **Frequency** |
| **Quantity** | - | - | - | - | - | - | - | - |
| **Frequency** | 0.72 | - | 0.56 | - | 0.41 | - | 0.47 | - |
| **DSM-5 criteria** | 0.54 | 0.54 | 0.47 | 0.52 | 0.32 | 0.50 | 0.40 | 0.51 |

# Table S4-1 Descriptive overview of the quantity of use with median and range and separately for the assessment years and for the three baseline group, i.e. the substance use disorder (SUD) group, the behavioral addiction (BA) group, and the control group.

|  | Baseline | | | Follow-up 1 year | | | Follow-up 2 years | | | Follow-up 3 years | | |
| --- | --- | --- | --- | --- | --- | --- | --- | --- | --- | --- | --- | --- |
|  | SUD | BA | control | SUD | BA | Control | SUD | BA | Control | SUD | BA | Control |
| n | 100 | 118 | 120 | 91 | 106 | 112 | 64 | 70 | 61 | 67 | 89 | 95 |
| Quantity of use at a typical occasion | Median  (range) | Median  (range) | Median  (range) | Median  (range) | Median  (range) | Median  (range) | Median  (range) | Median  (range) | Median  (range) | Median  (range) | Median  (range) | Median  (range) |
| Tobacco (cigarettes) | 2.5  (0-20) | 0  (0-5) | 0  (0-7) | 4  (0-20) | 0  (0-20) | 0  (0-8) | 4  (0-20) | 0  (0-5) | 0  (0-2) | 3  (0-30) | 0  (0-5) | 0  (0-10) |
| Alcohol  (gram ethanol) | 49.5  (0-193.5) | 27  (0-180) | 36  (0-121.5) | 45  (0-153) | 27  (0-135) | 27  (0-126) | 45  (0-315) | 31.5  (0-153) | 36  (0-99) | 45  (0-90) | 36  (0-157.5) | 36  (0-103.5) |
| Internet use (hours) | 0  (0-6) | 2  (0-10) | 0  (0-5) | 2  (0.2-10) | 2  (1-8) | 2  (0-6) | 5  (0-5) | 5  (3-5) | 5  (1-5) | 5  (0-5) | 5  (3-5) | 5  (0-5) |
| Computer gaming  (hours) | 0  (0-3) | 0  (0-5) | 0  (0-4) | 1  (0-9) | 1  (0-8) | 0  (0-5) | 0  (0-6) | 0  (0) | 0  (0) | 0  (0-5) | 0  (0-4) | 0  (0-3) |
| Gambling (hours) | 0  (0-6) | 0  (0-1) | 0  (0) | 0  (0-5) | 0  (0-4) | 0  (0) | 0  (0-5) | 0  (0-1) | 0  (0) | 0  (0-4) | 0  (0-3) | 0  (0) |
| Shopping (hours) | 0  (0-3) | 0  (0-1) | 0  (0) | 0  (0-3) | 0  (0-8) | 0  (0-5) | 0  (0-5) | 0  (0-3) | 0  (0-3) | 0  (0-7) | 0  (0-3) | 0  (0-4) |

# Table S4-2 Descriptive overview of the frequency of use with median and range and separately for the assessment years and for the three baseline group, i.e. the substance use disorder (SUD) group, the behavioral addiction (BA) group, and the control group.

|  | Baseline | | | Follow-up 1 year | | | Follow-up 2 years | | | Follow-up 3 years | | |
| --- | --- | --- | --- | --- | --- | --- | --- | --- | --- | --- | --- | --- |
|  | SUD | BA | Control | SUD | BA | Control | SUD | BA | Control | SUD | BA | Control |
| n | 100 | 118 | 120 | 91 | 106 | 112 | 64 | 70 | 61 | 67 | 89 | 95 |
| Frequency of use per week | Median  (range) | Median  (range) | Median  (range) | Median  (range) | Median  (range) | Median  (range) | Median  (range) | Median  (range) | Median  (range) | Median  (range) | Median  (range) | Median  (range) |
| Tobacco use | 4  (0-7) | 0  (0-7) | 0  (0-7) | 4  (0-7) | 0  (0-7) | 0  (0-7) | 4  (0-7) | 0  (0-7) | 0  (0-4) | 2  (0-7) | 0  (0-7) | 0  (0-7) |
| Alcohol use | 2  (0-7) | 0.5  (0-4) | 0.5  (0-4) | 2  (0-7) | 0.5  (0-4) | 0.5  (0-4) | 2  (0-7) | 0.5  (0-4) | 0.5  (0-4) | 2  (0-7) | 0.5  (0-7) | 0.5  (0-4) |
| Internet use | 0  (0-7) | 7  (0-7) | 0  (0-7) | 7  (0-7) | 7  (0.5-7) | 4  (0-7) | 0.5  (0-7) | 2  (0-7) | 0.5  (0-4) | 0.5  (0-7) | 0.5  (0-7) | 0.5  (0-7) |
| Computer gaming | 0  (0-7) | 0  (0-7) | 0  (0-7) | 0.25  (0-7) | 0.5  (0-7) | 0  (0-7) | 0.25  (0-7) | 0.38  (0-7) | 0  (0-7) | 0  (0-7) | 0.5  (0-7) | 0.25  (0-7) |
| Gambling | 0  (0-2) | 0  (0-2) | 0  (0) | 0  (0-0.5) | 0  (0-2) | 0  (0) | 0  (0-4) | 0  (0-0.5) | 0  (0-0.5) | 0  (0-4) | 0  (0-2) | 0  (0-2) |
| Shopping | 0  (0-2) | 0  (0-2) | 0  (0) | 0  (0-0.5) | 0  (0-2) | 0  (0-7) | 0  (0-2) | 0  (0-2) | 0  (0-0.5) | 0  (0-2) | 0  (0-2) | 0  (0-2) |

# Table S4-3 Descriptive overview of the number of DSM-5 criteria for addictive disorders with median and range and separately for the assessment years and for the three baseline group, i.e. the substance use disorder (SUD) group, the behavioral addiction (BA) group, and the control group.

|  | Baseline | | | Follow-up 1 year | | | Follow-up 2 years | | | Follow-up 3 years | | |
| --- | --- | --- | --- | --- | --- | --- | --- | --- | --- | --- | --- | --- |
|  | SUD | BA | Control | SUD | BA | Control | SUD | BA | Control | SUD | BA | Control |
| n | 100 | 118 | 120 | 91 | 106 | 112 | 64 | 70 | 61 | 67 | 89 | 95 |
| Number of (adapted) DSM-5 criteria for addictive disorders related to |  |  |  |  |  |  |  |  |  |  |  |  |
| Tobacco use | 2  (0-7) | 0  (0-1) | 0  (0-1) | 0  (0-6) | 0  (0-6) | 0  (0-4) | 0  (0-6) | 0  (0-4) | 0  (0) | 0  (0-6) | 0  (0-5) | 0  (0-4) |
| Alcohol use | 2  (0-6) | 0  (0-1) | 0  (0-1) | 0  (0-5) | 0  (0-5) | 0  (0-4) | 0  (0-4) | 0  (0-3) | 0  (0-1) | 0  (0-6) | 0  (0-4) | 0  (0-2) |
| Internet use | 0  (0-1) | 3  (0-9) | 0  (0-1) | 0  (0-6) | 1  (0-8) | 0  (0-3) | 0  (0-4) | 1  (0-6) | 0  (0-3) | 0  (0-6) | 1  (0-7) | 0  (0-5) |
| Computer gaming | 0  (0-1) | 0  (0-8) | 0  (0-1) | 0  (0-1) | 0  (0-8) | 0  (0-2) | 0  (0-8) | 0  (0-6) | 0  (0-3) | 0  (0-5) | 0  (0-6) | 0  (0-4) |
| Gambling | 0  (0-1) | 0  (0-2) | 0  (0-1) | 0  (0-1) | 0  (0-2) | 0  (0) | 0  (0-1) | 0  (0-1) | 0  (0) | 0  (0-2) | 0  (0) | 0  (0) |
| Shopping | 0  (0-1) | 0  (0-1) | 0  (0) | 0  (0) | 0  (0-2) | 0  (0-2) | 0  (0-2) | 0  (0) | 0  (0) | 0  (0) | 0  (0-6) | 0  (0-2) |

# Table S5 Correlation between substance- and non-substance-related addictive behavior outcomes according to the assessment years

|  |  | **Baseline** | | | **Follow-up 1 year** | | | **Follow-up 2 years** | | | **Follow-up 3 years** | | |
| --- | --- | --- | --- | --- | --- | --- | --- | --- | --- | --- | --- | --- | --- |
|  |  | **Substance-related** | | | **Substance-related** | | | **Substance-related** | | | **Substance-related** | | |
|  |  | **Quantity** | **Frequen-cy** | **DSM-5 criteria** | **Quantity** | **Frequency** | **DSM-5 criteria** | **Quantity** | **Frequen-cy** | **DSM-5 criteria** | **Quantity** | **Frequen-cy** | **DSM-5 criteria** |
| **Non-substance-related** | **Quantity** | -0.07 | -0.18 | -0.13 | 0.19 | 0.09 | 0.16 | 0.17 | 0.03 | 0.05 | 0.28 | 0.19 | 0.27 |
|  | **Frequency** | -0.11 | -0.19 | -0.17 | 0.05 | -0.04 | -0.02 | -0.03 | -0.04 | 0.04 | 0.10 | 0.01 | 0.15 |
|  | **DSM-5 criteria** | -0.20 | -0.29 | -0.27 | -0.04 | -0.11 | 0.00 | -0.04 | -0.09 | 0.03 | -0.06 | -0.06 | 0.11 |

# **Table S6** Overview of the tasks in the task battery to assess individual differences in executive functioning (Wolff et al., 2020)

| Task | | Description | Outcome measure |
| --- | --- | --- | --- |
| Inhibition | |  |  |
|  | Go-nogo | On each trial a fixation cross (750 ms) was followed by two vertically or horizontally arranged dots (500 ms). Participants were instructed to press the response key^a^ on "go" trials (dots vertically arranged), but to withhold the response on "no-go" trials (dots horizontally arranged). There were 280 "go" and 40 "no-go" trials. | IES (using “go” RT and "no-go" ER) |
|  | Stop signal | On each trial a fixation cross (750 ms) was followed by a left- or right-pointing arrow. There were 200 "go" and 40 "stop" trials. On "go" trials, the arrow was shown for 1000 ms and participants were instructed to press the response key^a^ corresponding to the direction of the arrow. On "stop" trials, the sideward-pointing arrow was replaced by an upward-pointing arrow after a variable stop-signal delay (SSD), and participants had to withhold the response. To achieve a stop-trial ER of approximately 50% for all participants, the SSD (initially 200 ms) was adapted after each "stop" trial by adding 50 ms for a (correct) non-response and subtracting 50 ms otherwise. | Stop-signal reaction time (SSRT), estimated according to the quantile method^b^ (Congdon et al. 2012) |
|  | Stroop | On each trial a fixation cross (750 ms) was followed by a stimulus (1000 ms) consisting of a row of one, two, three, or four identical digits from *1* to *4*. Number and denotation of digits were either congruent (80 trials) or incongruent (80 trials). Participants were instructed to respond according to the number of presented digits by pressing the leftmost key^a^ for one, the second-leftmost key for two, the second-rightmost key for three, and the rightmost key for four digits. | IES difference between "congruent" and "incongruent" trials |
| Shifting^c^ | |  |  |
|  | Number-letter | Throughout the task the screen was divided into four quadrants by a horizontal and a vertical line. On each trial a digit-letter pair (e.g., *2a* or *g9*) was shown for 3000 ms in one quadrant. Digits were either even or odd, and letters were either vowels or consonants. Participants were instructed to use two response keys^a^ to indicate whether the digit was even or odd (when the stimulus was shown above the horizontal line), or to indicate whether the letter was a vowel or a consonant (when the stimulus was below the line). "Switch" trials alternated with "no-switch" trials as the stimulus location was rotated in a clock-wise direction from trial to trial. There were 128 trials in total. | IES difference between "switch" and "no-switch" trials |
|  | Color-shape | On each trial a fixation cross (600 ms) was followed by a cue word (either *color* or *shape*; 200 ms), a blank-screen cue-stimulus interval (CSI; 200 ms), and a stimulus (1500 ms). The stimulus had the shape of either a circle or a triangle, and its color was either red or green. Participants were instructed to use two response keys^a^ to indicate whether the color was red or green if the cue was *color*, and whether the shape was a circle or triangle when the cue was *shape*. There were 80 trials in total. | IES difference between "switch" and "no-switch" trials |
|  | Category switch | On each trial a fixation cross (600 ms) was followed by a cue (either a cross or a heart; 200 ms), a blank-screen CSI with variable duration, and a stimulus word describing an object (1500 ms). There were two response keys^a^. When the cue was a cross, the instruction was to indicate whether the described object was smaller or larger than a soccer ball. When the cue was a heart, the instruction was to indicate whether the object was animate or inanimate. There were four blocks (each 32 trials) in this task: CSIs were short (200 ms) in the first and third block, and long (1000 ms) in the second and fourth block. For the current study, only the 64 short-CSI trials were analyzed. | IES difference between switch and no-switch trials |
| Updating | |  |  |
|  | 2-back | Throughout the task eight circles arranged around the screen center were shown. For the first 1500 ms of each trial all circles were empty. Then one of the circles was filled out for 500 ms, giving the impression that it flashed. Participants were instructed to press the right key^a^ when the flashing circle was the same one that had flashed two trials earlier (“yes” trials), and to press the left key otherwise ("no" trials). There were 40 “yes” and 120 “no” trials. | IES across all trials |
|  | Letter memory | On each of the 18 trials, a sequence of either five, seven, or nine consonants was shown in the screen center. A fixation cross was shown for 2000 ms before each letter, and each letter was shown for 2000 ms. Participants then had to enter the last three letters of the sequence on the keyboard. Speeded responses were not required. | ER across all trials |

|  | AX-CP | On each trial a fixation cross (500 ms) was followed by a red letter (300 ms), a blank screen (200 ms), three black distractor letters (300 ms each), another blank screen (200 ms), and a green letter (1000 ms). There were 150 trials in total. In 105 “AX” trials, the red letter was an *A* and the green letter was an *X*. In 15 “AY” trials, the red letter was an *A* and the green letter was a non-*X.* In 15 “BX” trials, the red letter was a non-*A* and the green letter was an *X*. In 15 “BY” trials, the red letter was a non-*A* and the green letter was a non-*X*. Participants had to enter a response as soon as the green letter was shown, and the instruction was to press the right key^a^ on “AX” trials and the left key otherwise. | IES (using “AX” RT and "BX ER) |
| --- | --- | --- | --- |
| *Note:* ms = milliseconds; IES = inverse efficiency score; RT = response time; ER = error rate.  ^a^Depending on the number of response keys in a task, response keys were ‘space’ (one key), ‘Y’ and 'M’ (two keys), or ‘Y’, ‘C’, ‘B’, and 'M’ (four keys). Note that the 'Y' and 'Z' keys are transposed on German compared to English-layout keyboards.  ^b^For each participant, all correct "go" trials were arranged in ascending order, and the RT whose quantile corresponded to the participant’s ER on "stop" trials was selected (e.g., the fifty-second percentile RT for an ER of 0.52). The SSRT was then calculated by subtracting the average SSD from the quantile RT.  ^c^All shifting tasks comprised two subtasks, and on each trial there was a cue to inform participants which subtask should be performed. Thus there were "switch" trials (where the task set was different from the preceding trial) and "no-switch" trials (where the task set was the same as in the preceding trial). | | | |

# Table S7 Descriptive statistics for the executive function task outcomes at baseline for all participants and separately for the substance use disorder (SUD) group, the behavioral addiction (BA) group, and the control group.

|  | All | SUD | BA | Control |
| --- | --- | --- | --- | --- |
| n | 338 | 100 | 118 | 120 |
| Outcomes |  |  |  |  |
| Go-nogo  Included^a^  M(SD) | n=338  394.55 ms (85.51) | n=100  392.82 ms (80.10) | n=118  400.24 ms (86.29) | n=120  387.58  (89.26) |
| Stop signal  Included^a^  M(SD) | n=325  182.08 ms (50.07) | n=97  175.01 ms (50.03) | n=113  185.84 ms (50.93) | n=115  184.35 ms (49.07) |
| Stroop^b^  Included^a^  M(SD) | n=335  62.94 ms  (37.04) | n=100  59.94  (39.73) | n= 117  61.34 ms  (35.61) | n=118  67.08 ms  (38.61) |
| Number-letter^b^  Included^a^  M(SD) | n=332  331.64 ms (203.09) | n=100  327.77 ms (195.98) | n=115  349.43 ms (212.98) | n=117  317.46 ms (199.50) |
| Color-shape^b^  Included^a^  M(SD) | n=334  108.77 ms (82.05) | n=99  105.81 ms (80.80) | n=115  109.88 ms (90.60) | n=120  110.15 ms (74.75) |
| Category switch^b^  Included^a^  M(SD) | n=327  81.26 ms  (87.19) | n=97  86.82 ms  (84.65) | n=113  82.35 ms  (90.61) | n=117  75.61 ms  (86.30) |
| 2-back  Included^a^  M(SD) | n=321  549.28 ms (171.85) | n=96  555.30 ms (178.95) | n=112  552.17 ms (160.03) | n=113  541.30 ms (178.13) |
| Letter memory^c^  Included^a^  M(SD) | n=338  0.43  (0.22) | n=100  0.42  (0.22) | n=118  0.46  (0.21) | n=120  0.42  (0.21) |
| AX-CPT^d^  Included^a^  M(SD) | n=336  367.76 ms (54.74) | n=99  375.32  (53.97) | n=117  366.84 ms (52.55) | n=119  362.37 ms (57.18) |
| *Note*. AX-CP = AX continuous performance.  ^a^Observations were excluded when the binomial probability of achieving the observed accuracy in the critical condition by random responding was >.05. For the stop signal task, lenient exclusion criteria proposed by Congdon et al. (2012) were applied.  ^b^Difference scores (Stroop interference and switch cost, respectively) were replaced with 0 when negative.  ^c^Accuracy scores were arcsine transformed to improve normality.  ^d^IESs could not be calculated for 2 participants who had no correct BX trials. These observations were assigned the post-trimming maximum (534 ms). | | | | |

# Table S8 Results of the group difference tests between the drop-outs at the 3-years follow-up (FU3) and completers in terms of task performance, addictive severity (symptoms, quantity, frequency), and the control variables age, gender, IQ, school graduation, and group membership at baseline.

|  | Completers | Drop-out at FU3 | Test statistics |
| --- | --- | --- | --- |
| Total n=338 | 251 (74%) | 87 (26%) |  |
| Task performance |  |  |  |
| Go-nogo | 391.33 (5.16) | 400.04 (10.28) | *t*=-.81, *p*=.42 |
| Stop signal | 183.21 (3.18) | 178.69 (51.46) | *t*=.70, *p*=.48 |
| Stroop | 63.31 (2.39) | 61.86 (3.80) | *t*=.31, *p*=.76 |
| Number-letter | 331.45 (13.17) | 332.20 (20.85) | *t*=-.03, *p*=.98 |
| Color-shape | 111.28 (5.13) | 101.30 (9.26) | *t*=.96, *p*=.34 |
| Category switch | 82.58 (5.50) | 77.26 (90.38) | *t*=.48, *p*=.63 |
| 2-back | 545.42 (10.83) | 560.53 (20.38) | *t*=-.69, *p*=.49 |
| Letter memory | .43 (.01) | .43 (.02) | *t*=.00, *p*=.99 |
| AX-CPT | 365.23 (3.31) | 375.18 (6.63) | *t*=-1.45, *p*=.15 |
| Addictive disorder severity |  |  |  |
| Quantity | 0.37 (0.26) | 0.41 (0.27) | *t*=1.24, *p*=.22 |
| Frequency | 6.59 (4.29) | 7.08 (4.87) | *t*=0.88, *p*=.38 |
| DSM-5 criteria | 2.90 (0-16) | 3.34 (0-13) | *z*=1.06, *p*=0.29 |
| Control variables |  |  |  |
| Age | 21.81 (.11) | 21.93 (.17) | *t*=.58, *p*=.56 |
| Female gender | 146 (58%) | 53 (62%) | χ2=.36, *p*=.55 |
| IQ | 105.02 (0.62) | 102.53 (1.01) | *t*=-2.03, *p*=.04 |
| School graduation ‘Gymnasium’ | 95 (39%) | 25 (29%) | χ2=2.97, *p*=.23 |
| Group at baseline  SUD  ND  Controls | 69 (27%)  88 (35%)  95 (38%) | 31 (36%)  30 (35%)  25 (29%) | χ2=2.97, *p*=.23 |

# TableS9 Data distributions characteristics of the outcome variables for the latent growth modelling per assessment year.

|  | Baseline | | | | Follow-up 1 year | | | | Follow-up 2 years | | | | Follow-up 3 years | | | |
| --- | --- | --- | --- | --- | --- | --- | --- | --- | --- | --- | --- | --- | --- | --- | --- | --- |
|  | MD (SD) | Range | Skew-ness | Kurto-sis | MD (SD) | Range | Skew-ness | Kurto-sis | MD (SD) | Range | Skew-ness | Kurto-sis | MD (SD) | Range | Skew-ness | Kurto-sis |
| Quantity^a^ | 0.38  (0.26) | 0 –  1.6 | 0.98 | 4.04 | 0.60  (0.35) | 0 – 2.5 | 1.09 | 5.19 | 0.67  (0.24) | 0.2 – 2.6 | 2.83 | 18.98 | 0.70  (0.24) | 0 – 2.5 | 1.83 | 9.47 |
| Frequency^a^ | 6.71  (4.44) | 0.3 – 21.3 | 0.48 | 3.06 | 9.25  (4.37) | 0.3 – 23 | 0.51 | 3.30 | 5.51  (4.11) | 0 – 20 | 0.85 | 3.07 | 5.60  (4.20) | 0 – 20 | 0.97 | 3.21 |
| DSM-5 criteria^a^ | 3.03  (2.73) | 0-16 | 1.41 | 5.95 | 2.10 (2.58) | 0-16 | 1.68 | 6.33 | 1.48 (2.09) | 0-11 | 2.05 | 7.86 | 2.12 (2.68) | 0-16 | 2.10 | 8.87 |
| ^a^ The possible value ranges for the outcomes were: quantity 0 to 6 (according to the rescaling from 0 to 1 and the 6 addictive behaviors), frequency 0 to 42 (according to the maximum of seven days per week and the 6 addictive behaviors), and DSM-5 criteria 0 to 64 (according to the maximum of 11 DSM-5 criteria (with the exception of gambling disorder with 9 criteria) and the 6 addictive disorders). | | | | | | | | | | | | | | | | |

# Figure S1 Individual trajectories (light grey) and mean trajectory (black) with 95% confidence interval (dark grey) of the outcomes quantity of use, frequency of use, and number of fulfilled DSM-5 criteria for addictive disorders from baseline (year 0) to the 3 year follow-up according to the three baseline group, i.e. the substance use disorder (SUD) group, the behavioral addiction (BA) group, and the control group (CG)


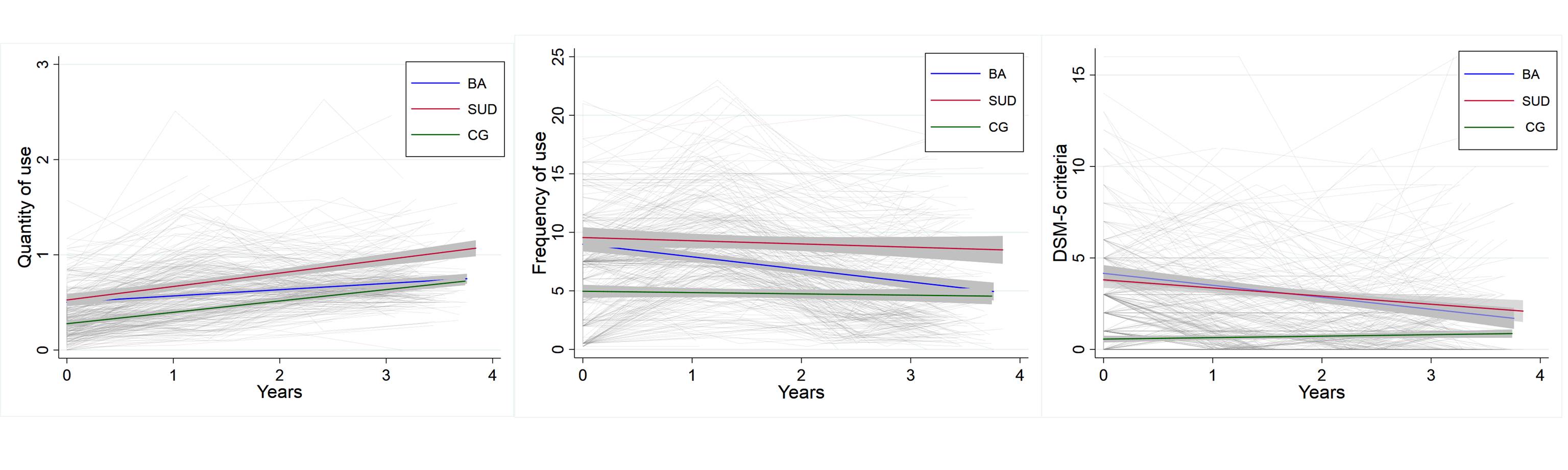


# Figure S2 Scatter plots on the negative associations between baseline latent general executive functioning (GEF) and the latent intercepts (A) and latent slopes (B) of the amount of use (left) and the frequency of use (right).


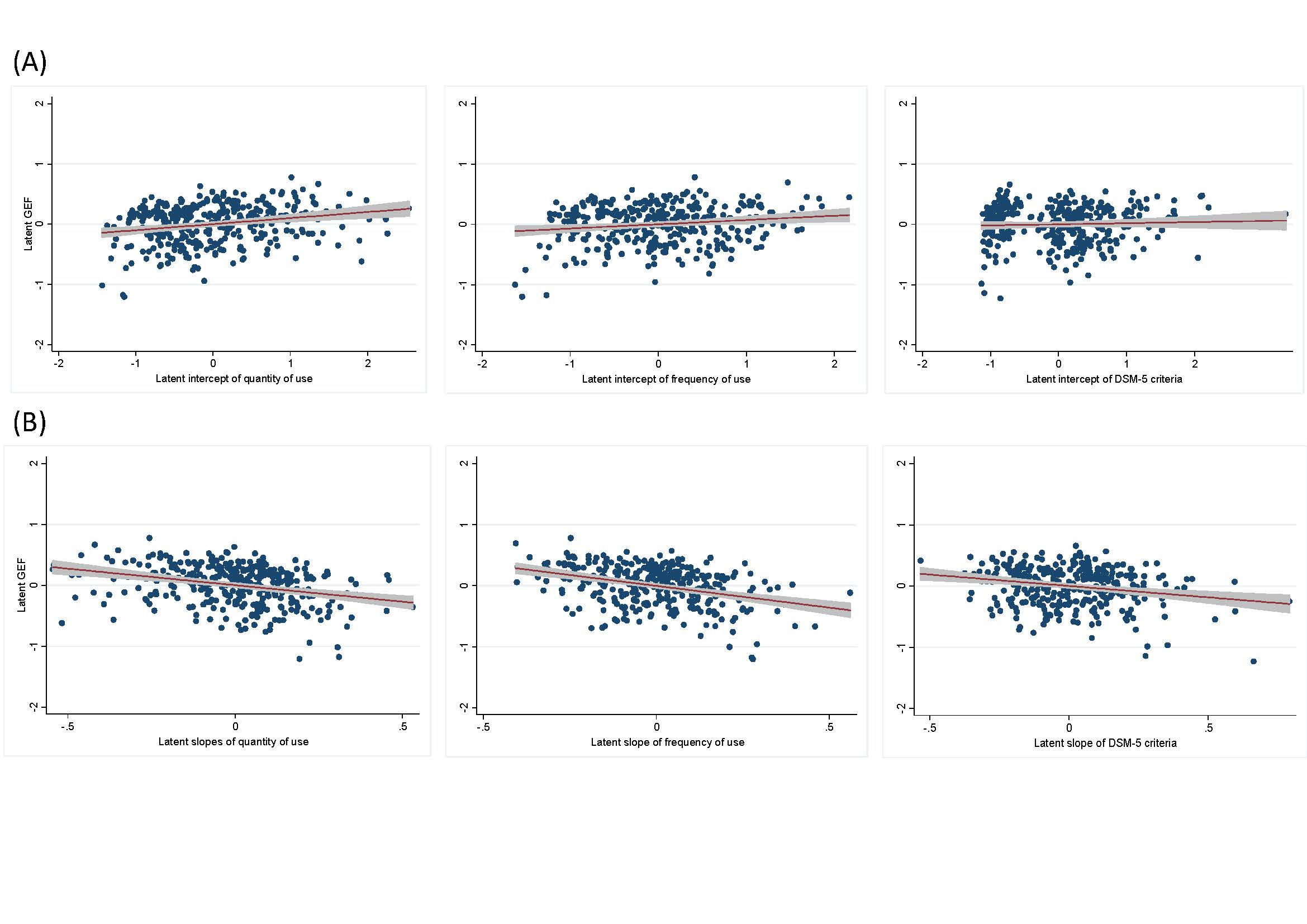


*Note:* The values are factor scores extracted from latent growth curve modelling.

# **Table S10** Results of the unadjusted conditional latent growth models testing the relationship between the quantity of use, frequency of use, and DSM-5 criteria for addictive disorders over time and predictor general executive functioning (GEF).

|  |  |  | Estimates^a^ | *p*-values | 95% confidence intervals |
| --- | --- | --- | --- | --- | --- |
| **Quantity of use** |  | Intercept | 0.32 | 0.06 | -0.02 – 0.67 |
|  |  | Slope | -0.13 | 0.05 | -0.26 – 0.00 |
|  |  |  |  |  |  |
| **Frequency of use** |  | Intercept | 0.27 | 0.11 | -0.06 – 0.60 |
|  |  | Slope | -0.13 | 0.04 | -0.27 – -0.002 |
|  |  |  |  |  |  |
| **DSM-5 addictive disorder criteria** | Second class of the ZIP model  (log count from Poisson part) | Intercept | -0.01 | 0.93 | -0.35 –0.33 |
|  |  | Slope | -0.25 | 0.18 | -0.72 – 0.23 |

^a^ standardized estimates

# **Table S11-1** Results of the exploratory latent growth models of the summed **quantity** of substance- and of non-substance-related use over time including the predictor general executive functioning (GEF).

|  | Predictor GEF | Estimates^a^ | *p*-value | 95% confidence interval |
| --- | --- | --- | --- | --- |
| Quantity of substance use (tobacco, alcohol) | Intercept | 0.06 | 0.70 | -0.25 – 0.38 |
|  | Slope | -0.05 | 0.39 | -0.17 – 0.06 |
|  | | | | |
| Quantity of non-substance-related use (Internet, gaming, gambling, shopping) | Intercept | 0.35 | 0.02 | 0.05 – 0.66 |
|  | Slope | -0.11 | 0.10 | -0.24 – 0.01 |
| Note: Time invariant control variables were baseline group membership and demographic characteristics (age, gender, IQ, and school graduation). | | | | |

^a^ standardized estimates

# **Table S11-2** Results of the exploratory latent growth models of the summed **frequency** of substance- and of non-substance-related use over time including the predictor general executive functioning (GEF).

|  |  | Estimates^a^ | *p*-value | 95% confidence interval |
| --- | --- | --- | --- | --- |
| Frequency of substance use (tobacco, alcohol) | Intercept | -0.005 | 0.97 | -0.31 – 0.30 |
|  | Slope | -0.06 | 0.27 | -0.18 – 0.05 |
|  | | | | |
| Frequency of non-substance-related use (Internet, gaming, gambling, shopping) | Intercept | 0.29 | 0.05 | -0.004 – 0.59 |
|  | Slope | -0.19 | 0.09 | -0.23 – 0.01 |
| Note: Time invariant control variables were baseline group membership and demographic characteristics (age, gender, IQ, and school graduation). | | | | |

^a^ standardized estimates

# **Table S11-3** Results of the exploratory latent growth models of the summed **DSM-5 criteria** for substance- and of non-substance-related addictive disorders over time including the predictor general executive functioning (GEF).

|  |  |  | **Estimate**s^a^ | ***p*-values** | **95% confidence intervals** |
| --- | --- | --- | --- | --- | --- |
| **DSM-5 substance use disorder criteria** | Second class of the ZIP model  (log count from Poisson part) | Intercept | -0.03 | 0.73 | -0.23 – 0.17 |
|  |  | Slope | -0.15 | 0.39 | -0.60 – 0.30 |
|  |  |  |  |  |  |
| **DSM-5 behavioral addiction criteria** | Second class of the ZIP model  (log count from Poisson part) | Intercept | 0.06 | 0.35 | -0.10 – 0.21 |
|  |  | Slope | -0.15 | 0.30 | -0.53 – 0.23 |

Note: Time invariant control variables were baseline group membership and demographic characteristics (age, gender, IQ, and school graduation).

^a^ standardized estimates

# **Table S12-1** Results of the supplementary latent growth models testing the relationship between the quantity of use over time and the predictor general executive functioning (GEF) separately for the substance-related disorder (SUD) group, the behavioral addiction (BA) group, and the control group.

|  |  | **Estimates^a^** | ***p*-value** | **95% confidence interval** |
| --- | --- | --- | --- | --- |
| **Substance-related quantity in SUD group** | Intercept | 0.67 | 0.13 | -0.21 – 1.55 |
|  | Slope | -0.27 | 0.09 | -0.59 – 0.04 |
|  | | | | |
| **Non-substance-related quantity in BA group^b^** | Intercept | 0.34 | 0.20 | -0.18 – 0.87 |
|  | Slope | -0.15 | 0.22 | -0.40 – 0.09 |
|  | | | | |
| **Overall quantity in the control group** | Intercept | 0.61 | 0.05 | -0.01 – 1.24 |
|  | Slope | -0.13 | 0.23 | -0.35 – 0.08 |
| Note: Time invariant control variables were age, gender, IQ, and school graduation. | | | | |

^a^ standardized estimates

^b^ Due to the small sample size, the model could only be properly calculated without the control variables.

# **Table S12-2** Results of the supplementary latent growth models testing the relationship between the frequency of use over time and the predictor general executive functioning (GEF) separately for the substance-related disorder (SUD) group, the behavioral addiction (BA) group, and the control group.

|  |  | **Estimates^a^** | ***p*-value** | **95% confidence interval** |
| --- | --- | --- | --- | --- |
| **Substance-related frequency in SUD group** | Intercept | 0.20 | 0.65 | -0.67 – 1.07 |
|  | Slope | -0.38 | 0.03 | -0.71 – -0.04 |
|  | | | | |
| **Non-substance-related frequency in BA group** | Intercept | 0.43 | 0.18 | -0.19 – 1.04 |
|  | Slope | -0.19 | 0.73 | -1.25 – 0.88 |
|  | | | | |
| **Overall frequency in the control group** | Intercept | 0.33 | 0.14 | -0.10 – 0.76 |
|  | Slope | -0.04 | 0.60 | -0.19 – 0.11 |
| Note: Time invariant control variables were age, gender, IQ, and school graduation. | | | | |

^a^ standardized estimates

# **Table S12-3** Results of the supplementary latent growth models testing the relationship between the DSM-5 criteria for addictive disorders over time and the predictor general executive functioning (GEF) separately for the substance-related disorder (SUD) group, the behavioral addiction (BA) group, and the control group.

|  |  | **Estimates^a^** | ***p*-value** | **95% confidence interval** |
| --- | --- | --- | --- | --- |
| **Substance-related DSM-5 criteria in SUD group** | Intercept | 0.13 | 0.81 | -0.88 – 1.13 |
|  | Slope | -0.27 | 0.30 | -0.79 – 0.24 |
|  | | | | |
| **Non-substance-related DSM-5 criteria in BA group** | Intercept | 0.28 | 0.25 | -0.20 – 0.76 |
|  | Slope | -0.02 | 0.82 | -0.14 – 0.11 |
|  | | | | |
| **Overall DSM-5 criteria in the control group** | Intercept | 0.26 | 0.28 | -0.21 – 0.72 |
|  | Slope | -0.22 | 0.18 | -0.54 – 0.10 |
| Note: Time invariant control variables were age, gender, IQ, and school graduation. | | | | |

^a^ standardized estimates

# Supplementary references

Congdon E, Mumford JA, Cohen JR, Galvan A, Canli T, Poldrack RA (2012) Measurement and reliability of response inhibition. Front Psychol 3:1-10. <https://doi.org/10.3389/fpsyg.2012.00037>

Fan X, Fan X (2005) Power of latent growth modeling for detecting linear growth: Number of measurements and comparison with other analytic approaches. The Journal of experimental education 73:121-139. <https://doi.org/>

Goudriaan AE, Oosterlaan J, de Beurs E, van den Brink W (2005) Decision making in pathological gambling: a comparison between pathological gamblers, alcohol dependents, persons with Tourette syndrome, and normal controls. Cognitive Brain Research 23:137-151. <https://doi.org/10.1016/j.cogbrainres.2005.01.017>

Goudriaan AE, Oosterlaan J, de Beurs E, van den Brink W (2006) Neurocognitive functions in pathological gambling: a comparison with alcohol dependence, Tourette syndrome and normal controls. Addiction 101:534-547. <https://doi.org/10.1111/j.1360-0443.2006.01380.x>

Gustavson DE, Stallings MC, Corley RP, Miyake A, Hewitt JK, Friedman NP (2017) Executive functions and substance use: Relations in late adolescence and early adulthood. JAP 126:257-270. <https://doi.org/10.1037/abn0000250>

Wolff M, Enge S, Kräplin A, Krönke KM, Bühringer G, Smolka MN, Goschke T (2020) Chronic stress, executive functioning, and real‐life self‐control: An experience sampling study. J Pers10.1111/jopy.12587
